# Picomolar fluorescent probes for compound affinity determination to carbonic anhydrase IX expressed in live cancer cells

**DOI:** 10.1038/s41598-022-22436-1

**Published:** 2022-10-21

**Authors:** Jurgita Matulienė, Gediminas Žvinys, Vytautas Petrauskas, Agnė Kvietkauskaitė, Audrius Zakšauskas, Kirill Shubin, Asta Zubrienė, Lina Baranauskienė, Lina Kačenauskaitė, Sergei Kopanchuk, Santa Veiksina, Vaida Paketurytė-Latvė, Joana Smirnovienė, Vaida Juozapaitienė, Aurelija Mickevičiūtė, Vilma Michailovienė, Jelena Jachno, Dovilė Stravinskienė, Aistė Sližienė, Agnė Petrošiūtė, Holger M. Becker, Justina Kazokaitė-Adomaitienė, Ala Yaromina, Edita Čapkauskaitė, Ago Rinken, Virginija Dudutienė, Ludwig J Dubois, Daumantas Matulis

**Affiliations:** 1 Department of Biothermodynamics and Drug Design, Institute of Biotechnology, Life Sciences Center, Vilnius University, Saulėtekio 7, LT-10257 Vilnius, Lithuania; 2 Latvian Institute of Organic Synthesis, Aizkraukles 21, Riga, LV-1006 Latvia; 3 Institute of Chemistry, University of Tartu, Ravila 14a, 50411 Tartu, Estonia; 4 Department of Immunology and Cell Biology, Institute of Biotechnology, Life Sciences Center, Vilnius University, Saulėtekio 7, LT-10257 Vilnius, Lithuania; 5 Zoology and Animal Physiology, Institute of Zoology, TU Dresden, 01217 Dresden, Germany; 6 The M-Lab, Department of Precision Medicine, GROW – School for Oncology and Reproduction, Maastricht University, Universiteitssingel 50/23, 6200 MD Maastricht, The Netherlands; 7 Present Address: Division of Biochemistry, The Netherlands Cancer Institute, Amsterdam, The Netherlands

**Keywords:** Biophysics, Cancer, Chemical biology

## Abstract

Numerous human cancers, especially hypoxic solid tumors, express carbonic anhydrase IX (CAIX), a transmembrane protein with its catalytic domain located in the extracellular space. CAIX acidifies the tumor microenvironment, promotes metastases and invasiveness, and is therefore considered a promising anticancer target. We have designed a series of high affinity and high selectivity fluorescein-labeled compounds targeting CAIX to visualize and quantify CAIX expression in cancer cells. The competitive binding model enabled the determination of common CA inhibitors’ dissociation constants for CAIX expressed in exponentially growing cancer cells. All tested sulfonamide compounds bound the proliferating cells with similar affinity as to recombinantly purified CAIX. The probes are applicable for the design of selective drug-like compounds for CAIX and the competition strategy could be applied to other drug targets.

## Introduction

Since the discovery that carbonic anhydrase isozyme IX (CAIX) is highly overexpressed in numerous cancers^1–3^, especially in hypoxic solid tumors, the enzyme has become a target of anticancer drug design. CAIX is a transmembrane protein that catalyzes the reversible hydration of CO_2_ into a bicarbonate anion and an acid proton, acidifies tumor cell microenvironment, and promotes metastasis and invasiveness^4–6^. Humans have 15 CA isozymes, where CAI, CAII, CAIII and CAVII are cytosolic, CAIX, CAXII and CAXIV—transmembrane, CAIV—attached to membrane via a lipid residue, CAVI—secreted, CAVA and CAVB—mitochondrial. CAVIII, CAX, and CAXI are catalytically inactive isozymes because the Zn(II) is absent in their active site^7^.

In healthy humans, CAIX is primarily expressed in the stomach and testes. However, its expression increases dramatically in numerous cancers, including cervical carcinoma, esophageal carcinoma, pancreatic tumor, kidney carcinoma, endometrial adenocarcinoma, ovarian tumor, urinary bladder carcinoma, colon adenocarcinoma, lung tumor, liver carcinoma, breast adenocarcinoma, squamous cell carcinoma of the head and neck, and others^8,9^. Compounds that would exhibit high affinity and selectivity for CAIX would have a promising therapeutic potential. Furthermore, the CAIX-selective fluorescent^10,11^ or PET^12^ probes could visualize the tumor for optically guided surgery or patient stratification.

We have designed and evaluated a series of compounds that bind CAIX with 10 pM to 50 pM affinities and showed 30 to 100,000-fold selectivities over other CA isozymes^13–18^. To possess high affinity, fluorines had to be introduced into the benzene ring directly attached to the sulfonamide head-group that binds the Zn(II) in the active site of CA. These fluorines withdraw electrons from the sulfonamide group and diminish its p*K*_*a*_ leading to an increase in affinity. The compound selectivity for CAIX is increased by introducing a bulky hydrophobic group as a *meta* substituent in the benzene ring relative to the sulfonamide group. However, these compounds were only shown to bind the purified CAIX selectively but have not been demonstrated to recognize CAIX in the cellular environment in the presence of thousands of other competing proteins.

Since the demonstration by Whitesides and coworkers that various substituents can be introduced in CA sulfonamide inhibitors, reviewed in^19^, various fluorescent probe-labeled sulfonamides have been synthesized. Naphthalene sulfonamide was used as a fluorogenic group^20^, fluorescein was attached to *para*- or *meta*- substituted benzene sulfonamide or acetazolamide^10,11,21–23^. Rhodamine^24^ and infrared dyes were attached to acetazolamide^9,25–27^ to image carbonic anhydrases, especially to follow CAIX expression in cell cultures and mice tumors. Fluorescent dye displacement assays have been used to determine inhibitor efficacy for bovine CAII^19,28^.

Here we have designed a series of compounds by conjugating fluorescein to CAIX-selective high-affinity inhibitors. Fluorescein was attached to VD11-4-2 with high affinity for CAIX and also other compounds that possess average or low affinities for CAIX as controls to account for non-specific binding. These fluorescent compounds not only helped to visualize CAIX expression in cancer cells but also became the basis of a technique to determine dissociation constants of common non-fluorescent CAIX inhibitors via competition with fluorescein-labeled compounds. We demonstrated that the tested inhibitors exhibited comparable affinities between recombinantly produced CAIX and the CAIX on the cell surface of live cancer cells. The dynamics of CAIX appearance on cancer cells was visualized and quantified. Super-resolution microscopy demonstrated the co-localization of fluorescently-labeled compounds with the CAIX antibodies.

## Results

### Design and affinity of fluorescein-labeled compounds that specifically recognize CAIX

**Figure 1:**
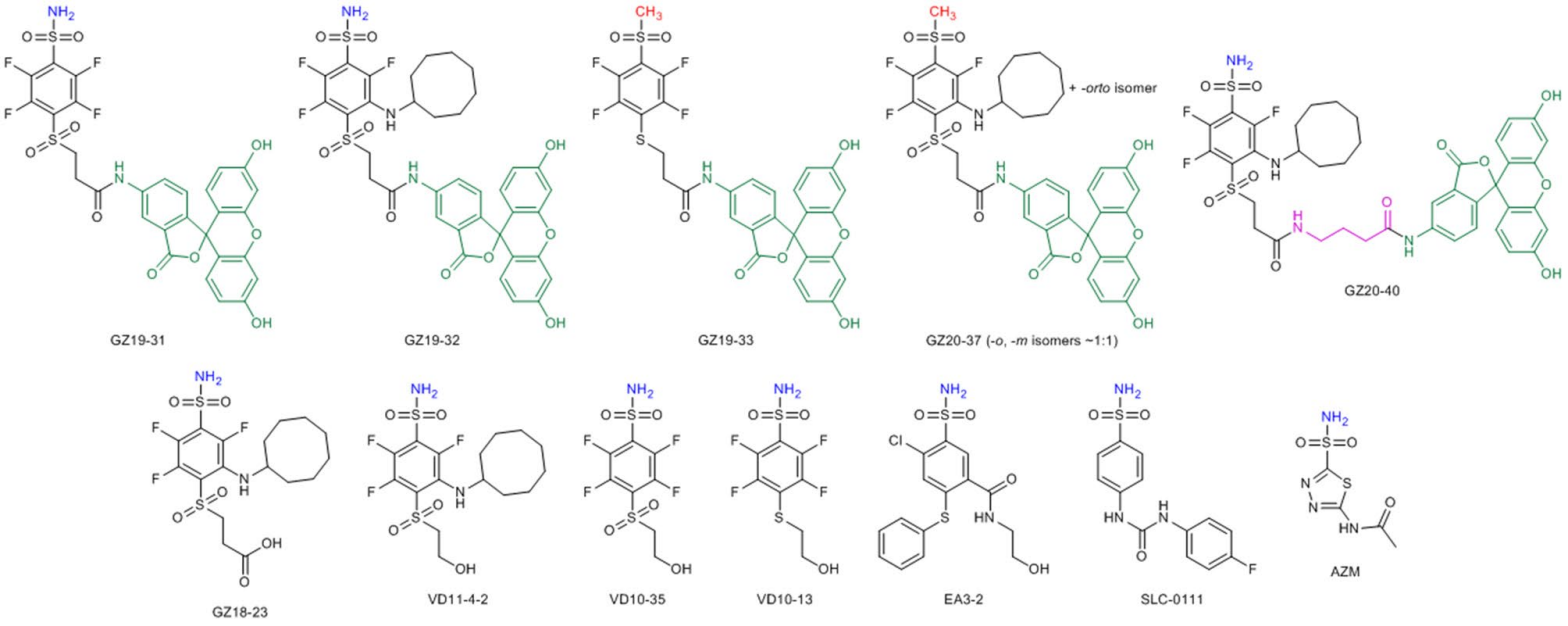
Chemical structures of fluorescein-labeled compounds (upper row) and several unlabeled compounds (lower row). Their affinities for CAIX on the cell surface were measured by the competition assay (described below). The amine of the sulfonamide group is shown in blue, while the methyl for non-inhibitors—in red. Fluorescein is shown in green and the extended linker—in pink.

A series of our previously designed compounds, which have been demonstrated to bind human CAIX with high affinity (*K*_d_ ~ 30 pM) and high selectivity over the remaining 11 catalytically active human CA isozymes^14^, were labeled with fluorescein as described in the methods part. The chemical structures of all compounds used in this study are shown in Fig. 1. To prepare the lead compound GZ19-32, fluorescein was conjugated to the tail of the VD11-4-2 molecule, exposed towards the solvent as shown by crystallography^16^.

Since there was a concern that fluorescein presence on a short linker may hinder the binding of such a conjugate to CAIX, we synthesized another compound, GZ20-40, bearing a longer linker between the fluorescein and VD11-4-2 (Fig. 1). Furthermore, we synthesized GZ19-31, a molecule with fluorescein attached to VD10-35, which has a high affinity for CAI and relatively low affinity for CAIX.

In addition, two control compounds, GZ20-37 and GZ19-33, were synthesized identical to the compounds GZ19-32 and GZ19-31, respectively, except that they contained a methyl group instead of an amino group in the sulfonamide headgroup. Both control compounds were expected to have low affinity for any CA isozyme, including recombinant CAIX and endogenous CAIX expressed on the cell surface.

The binding affinities of all fluorescein-containing compounds were determined for all 12 catalytically active CA isozymes by the thermal shift assay (TSA). In addition, we used the stopped-flow assay (SFA) to determine whether the compounds inhibited the catalytic activity of CA isozymes. However, the SFA technique cannot determine the affinities lower than the enzyme concentration used in the assay. Since we used 10–50 nM concentrations of CA isozymes, the SFA could not determine the affinities in the pM range. In addition, we used isothermal titration calorimetry (ITC), the standard assay to determine affinity and enthalpy of protein–ligand binding. However, direct ITC has a limitation that it can determine affinities up to approximately 10 nM *K*_d_. Therefore, ITC was used only to determine the enthalpy of interaction and confirm the ordering of compound affinities.

**Figure 2:**
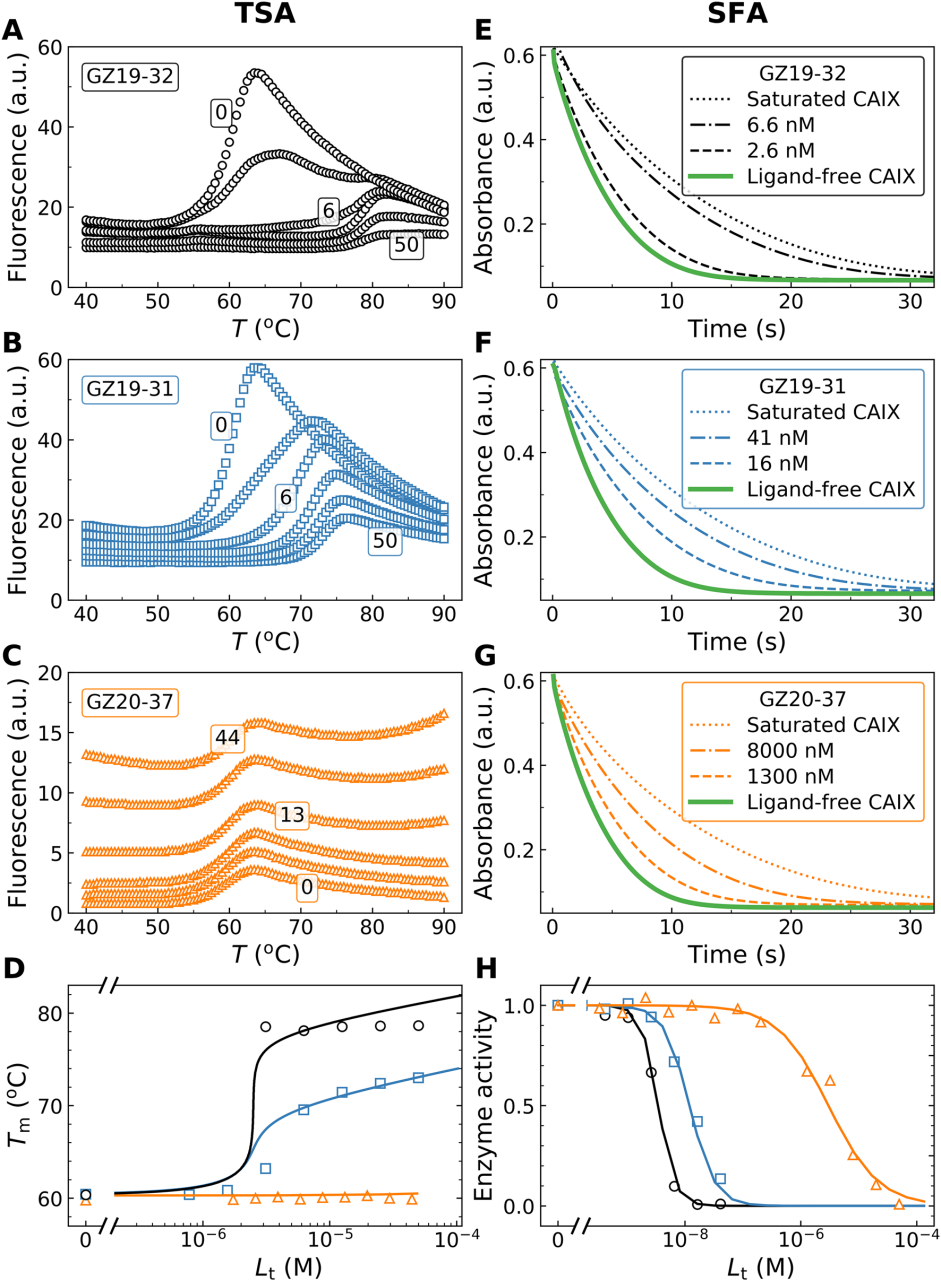
Determination of fluorescein-labeled compound affinities for the catalytic domain of recombinant human CAIX by the TSA (left panels) and the SFA (right panels). The upper three rows show the raw binding data for GZ19-32 (black), GZ19-31 (blue), and GZ20-37 (orange). (**A**, **B**, and **C**) The fluorescence profiles upon protein thermal melting. (**E**, **F**, and **G**) The absorbance decrease due to pH indicator color change upon acidification by CAIX in the SFA assay. The ‘ligand saturated CAIX’ dotted line matches the line observed for the spontaneous hydration of CO_2_. The bottom panels show the dosing curves by TSA (**D**) and SFA (**H**). SFA is limited to the affinity which is close to the concentration of CA used in the enzymatic assay. Therefore, only the TSA yielded accurate *K*_d_ values in the pM affinity range.

According to our TSA results, GZ19-32 possessed high affinity for CAIX (pM range). The CAI-selective GZ19-31 had a medium affinity (nM range) for CAIX (Fig. 2). The same order was confirmed by ITC (Figure S1). The binding affinity of GZ19-31 was comparable to the affinity determined by TSA and SFA. The methylated analogous control compound GZ20-37 did not show binding to CAIX by TSA or ITC, but weakly inhibited the CAIX activity (*K*_i_ = 1.5 µM). Compound affinities for the recombinantly purified catalytic domain of human CAIX and other isoforms are listed in Table 1. We concluded that the fluorescein-labeled GZ19-32 binds recombinant CAIX with the same affinity as unlabeled VD11-4-2 and GZ18-23. Thus the addition of fluorescein label did not affect the binding of the lead compound and GZ19-32 can be used as a reporter selective for CAIX.

**Table 1:** Dissociation constants of the fluorescein-labeled CA inhibitors and non-labeled inhibitors (chemical structures shown in Fig. 1).

Compound	*K*_d_ (nM)											
	CAI	CAII	CAIII	CAIV	CAVA	CAVB	CAVI	CAVII	CAIX	CAXII	CAXIII	CAXIV
**Fluorescein-labeled compounds**												
GZ19-31	0.0050	1.7	2500	140	29	20	140	2.0	1.7	40	1.7	25
GZ19-32	500	14	1670	100	400	13	330	5.0	0.005	1.3	0.67	3.3
GZ20-40	500	200	6700	500	1000	33	250	7.0	0.010	7.0	1.0	2.5
GZ20-37	>1·10^5^	>1·10^5^	>1·10^5^	>1·10^5^	>1·10^5^	>1·10^5^	>1·10^5^	>1·10^5^	>1·10^5^	>1·10^5^	>1·10^5^	>1·10^5^
GZ19-33	>1·10^5^	>1·10^5^	>1·10^5^	>1·10^5^	>1·10^5^	>1·10^5^	>1·10^5^	>1·10^5^	>1·10^5^	>1·10^5^	>1·10^5^	>1·10^5^
**Unlabeled compounds**												
VD11-4–2*	830	56	34,000	61	3300	16	67	8.6	0.032	2.9	4.0	4.3
GZ18-23	5000	160	>2·10^5^	120	12,500	160	140	43	0.033	1.4	10	3.3
VD10-35*	0.20	17	20,000	510	310	22	67	7.1	41	250	30	33
VD10-13*	0.15	11	29,000	440	400	1.7	200	13	32	220	11	5.0
EA3-2*	10,000	31	15,000	1.4	5600	330	1400	11	4.0	6.2	100	3.0
SLC-0111*	1100	330	> 2·10^5^	3100	6000	430	10,000	250	100	5500	2200	270
AZM*	2400	46	40,000	87	840	140	220	13	21	130	120	63

We also determined the affinities of the commonly used inhibitors SLC-0111 and acetazolamide (AZM) as controls of the assay. The dissociation constants are the *observed* ones (not *intrinsic*), given in nM units, determined by the TSA for 37 °C at pH 7.0

*The *K*_d_ values for VD11-4-2, VD10-35, VD10-13, EA3-2, and AZM were taken from^29^. The *K*_d_ values for SLC-0111 binding to CAII and CAXII were taken from^30^.

### Microscopy of CAIX-expressing human cancer cells stained by fluorescein-labeled compounds

**Figure 3:**
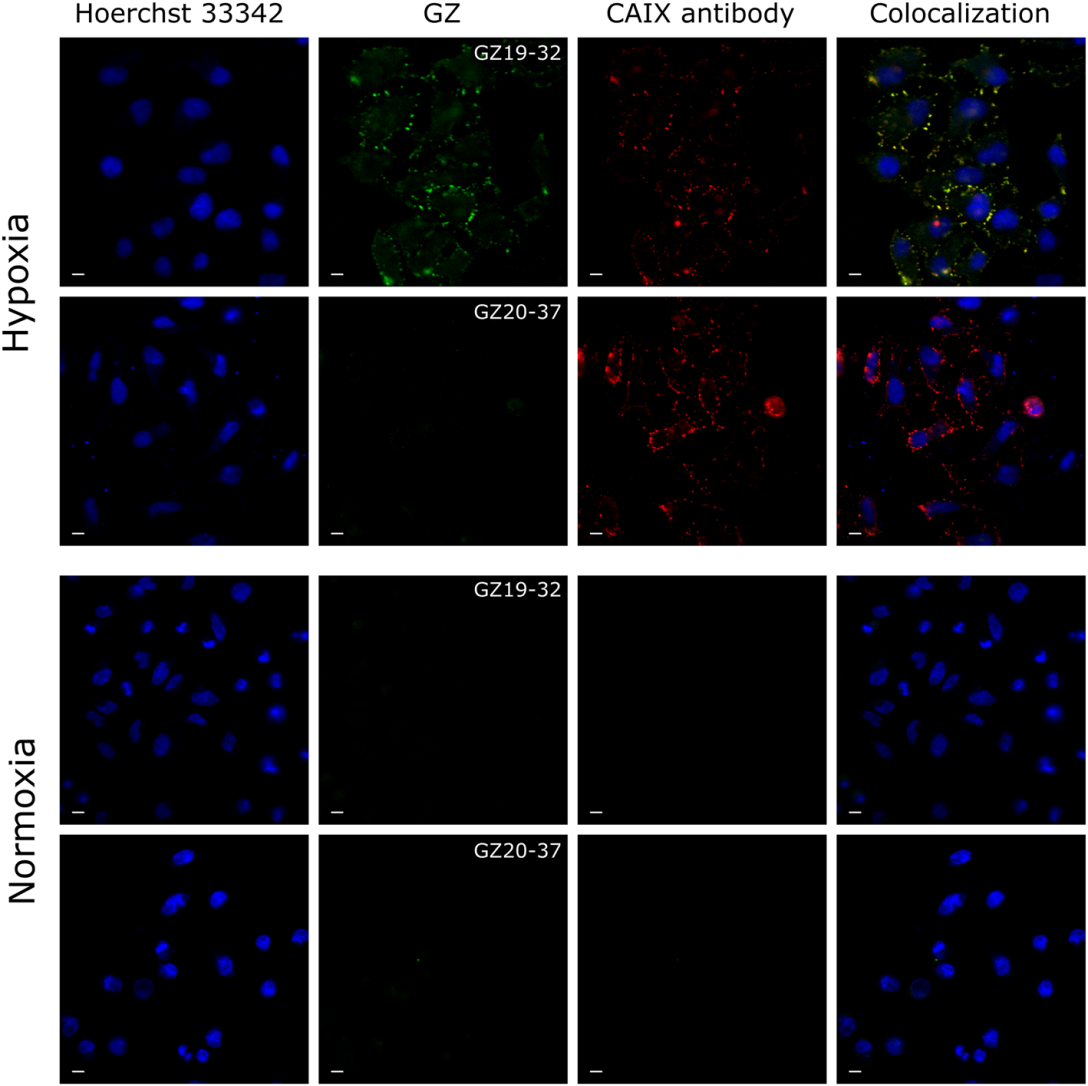
Staining of live HeLa cells (grown under hypoxia or normoxia) incubated with 10 nM GZ19-32 or GZ20-37 showing cell nuclei (Hoechst 33342, blue), binding of the GZ compounds (green), CAIX antibody (red) and their colocalization. The scale bar length is 10 µm.

To visualize the binding of fluorescein-labeled compounds to live cells, we performed microscopy of cells incubated with GZ19-32 (Fig. 3, Figure S2). The CAIX-selective GZ19-32 labeled the cell membrane, well seen in confluent cells (left upper panel compared to the fourth upper panel, Figure S2), while the methylated GZ20-37 did not bind to the cells under the same conditions (Figure S2, Fig. 3). The third column in Fig. 3 shows cell staining with the monoclonal CAIX antibody H7^31^. Staining by GZ19-32 perfectly co-localized with the staining by the antibody (fourth column). The first column shows the staining of DNA in the nuclei. This was a direct visual determination of the specific compound binding and staining of CAIX expressed on cancer cell membrane under hypoxia.

**Figure 4:**
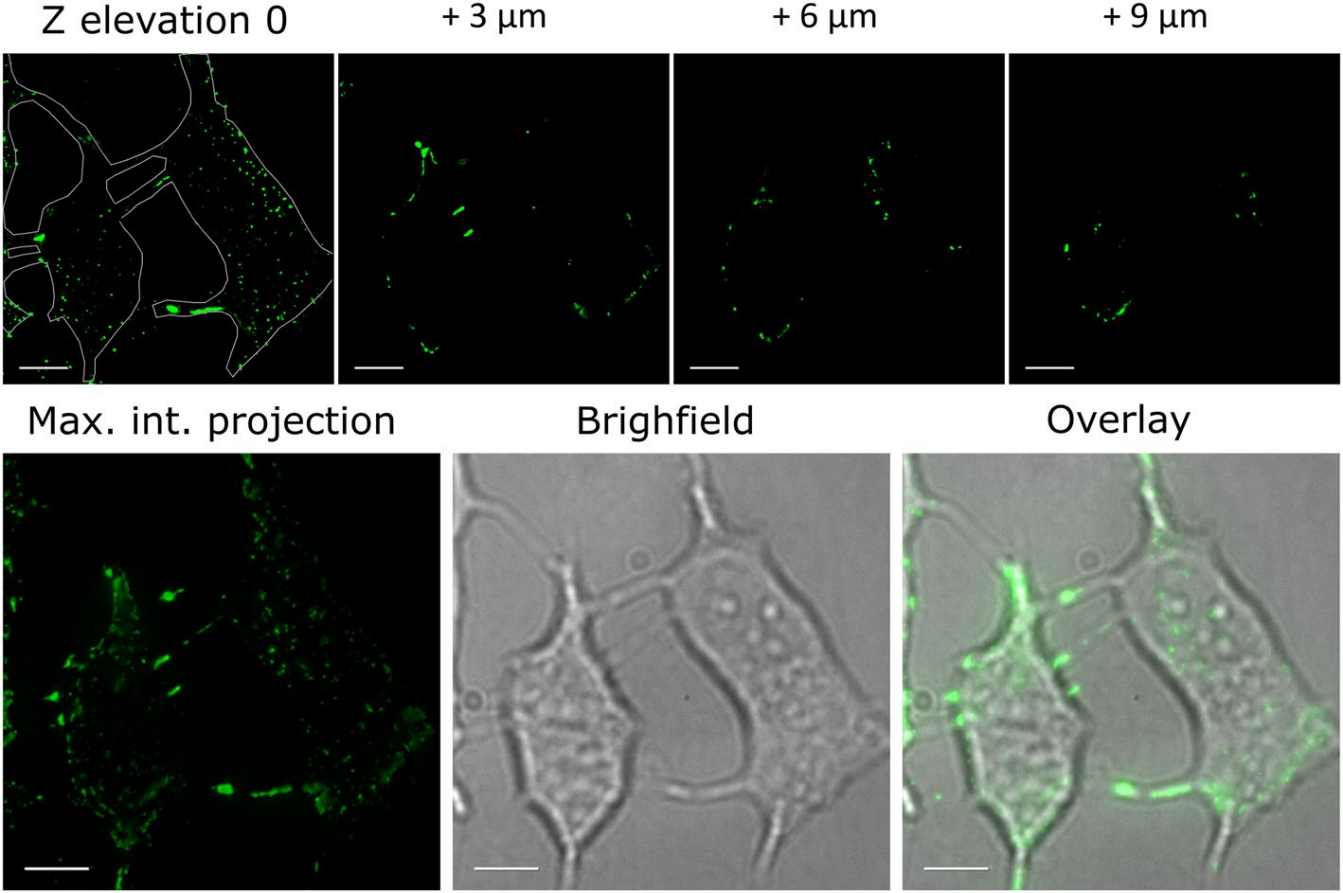
Localization of GZ19-32 (10 nM, green) in hypoxia-grown live HeLa cells shows that CAIX is expressed on the cell membrane, primarily on philopodia. The scale bar length is 10 µm.

**Figure 5:**
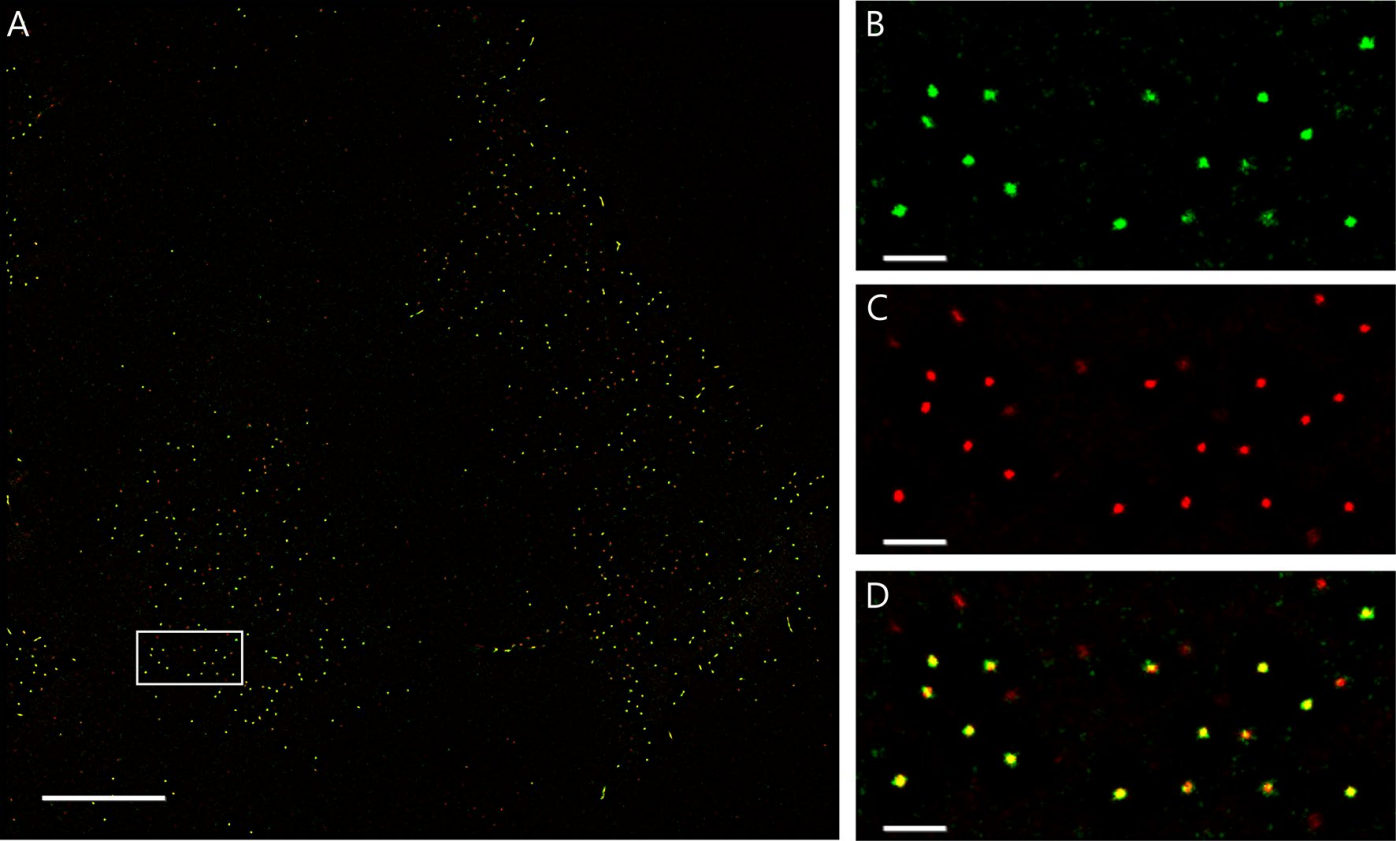
Here, the same two cells as in Fig. 4, are shown at super-resolution, visualized by SRRF microscopy with TIRF illumination and illustrate the positioning of the GZ19-32 (green, (**B**), CAIX antibody (**C**), and their colocalization (**D**). The scale bar is 10 µm in (**A**) and 1 µm in (**B**), (**C**), and (**D**).

In the individual cells, the fluorescein-labeled GZ19-32 was seen to localize to philopodia and threads connecting the cells and also other places on the cell membrane indicating localization of CAIX (Fig. 4). Super-Resolution Radial Fluctuations (SRRF) microscopy in TIRF mode illumination showed GZ19-32 in well-distinct spots that contain at least 10 molecules of CAIX per agglomerate (conservative estimations due to the observed smooth photobleaching time-traces without clear step-like behavior, Fig. 5). The GZ19-32 fluorescein-labeled CAIX-selective dye formed the green spots, while the same localization of the H7 antibody was visible in the red channel. The overlay indicated that the compound and the antibody were co-localized nearly perfectly. All used multicolor collocalization techniques indicated high degree of colocalization, namely, the pixel based Person’s correlation coefficient was 0.82 and Manders overlap coefficients were M1 0.82 and M2 0.65, and object based spot collocalization was 0.75. The size of each spot was smaller than 100 nm in diameter and were well-separated by similar distances of 0.7 to 1.8 µm. Similar observations were obtained with smaller concentration of GZ19-32 (2 nM, Figure S3).

The colocalization between GZ19-32 and CAIX was also demonstrated with another CAIX-specific monoclonal antibody M75 (Figure S4 A,B). In contrast, no yellow colocalization spots were seen when the antibody 1B10, specific for another membrane-associated isozyme CAXII ^32^, was used (Figure S4 C,D), or when the primary antibody was omitted in the staining procedure (Figure S4 E,F).

### Generation and validation of CRISPR-Cas9 CAIX-knockout HeLa cell line

**Figure 6:**
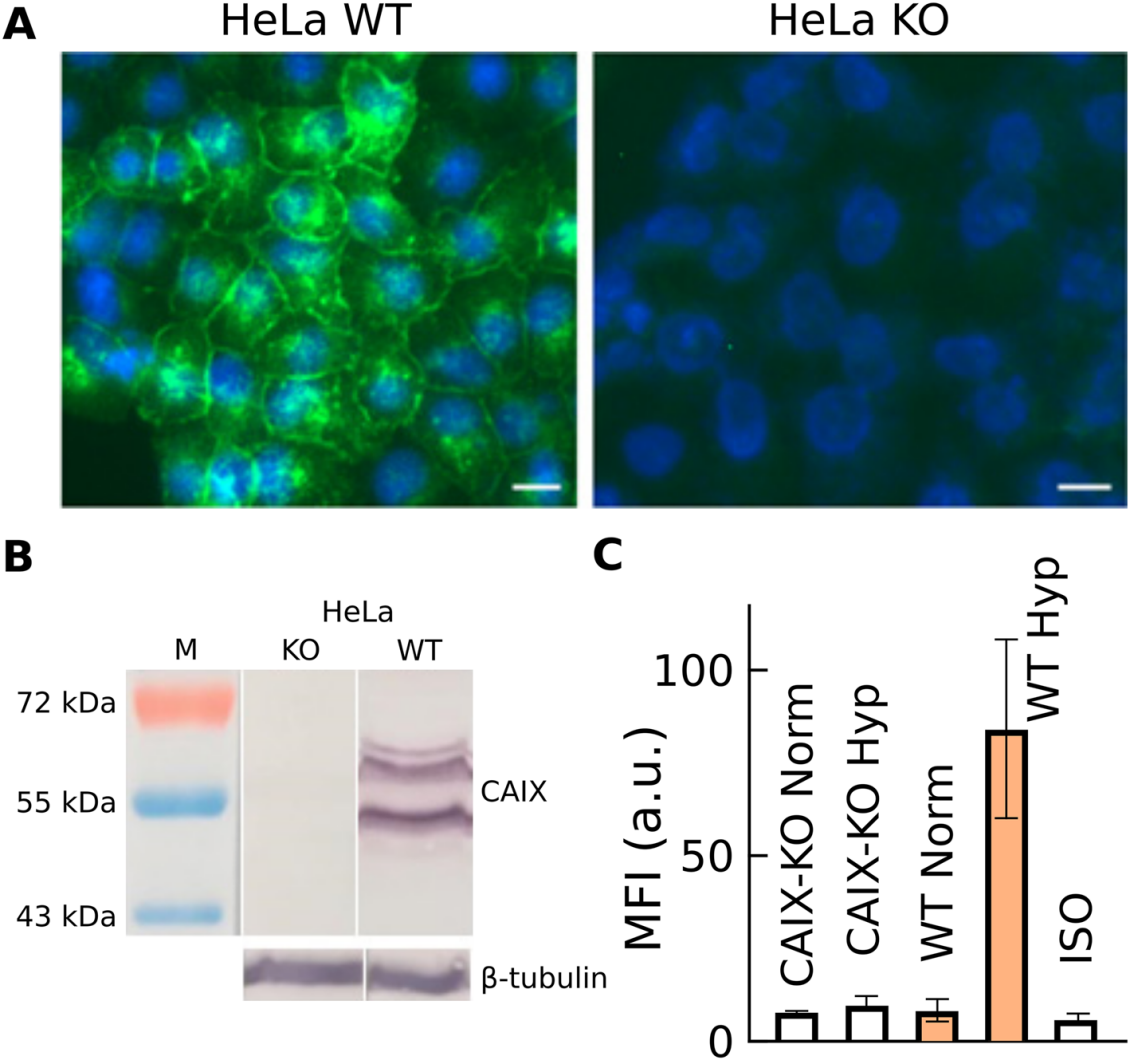
Validation of the CAIX knockout HeLa cells. (**A**) Immunofluorescence staining with H7 antibody of HeLa WT and HeLa CAIX-KO cells grown under hypoxia. Green staining of CAIX-expressing cell membranes is visible only in the WT cells. (**B**) Western Blot of HeLa WT and HeLa CAIX-KO hypoxia-grown cells using M75 monoclonal antibody and anti β-tubulin antibody as the loading control, demonstrated the absence of CAIX expression in the KO cells. The full-length membrane is shown in supplementary figure S5. (**C**) The mean fluorescence intensity (MFI) obtained by the flow cytometry of Hela WT or Hela CAIX-KO cell lines grown under normoxia or hypoxia. Average values from two experiments are shown, indicating CAIX presence only in the WT HeLa cells grown only under hypoxia.

To further study the binding and selectivity of designed fluorescent compounds to the cellular CAIX, we decided to knock-out CAIX in cancer cells. The CRISPR-Cas9 methodology was applied to generate the CAIX-knockout HeLa cells (CAIX-KO) as described in the methods part. The CAIX knockout in HeLa cells was confirmed by sequencing and immunofluorescence cell staining, Western blot (WB), and flow cytometry using antibodies for CAIX (Fig. 6).

**Figure 7:**
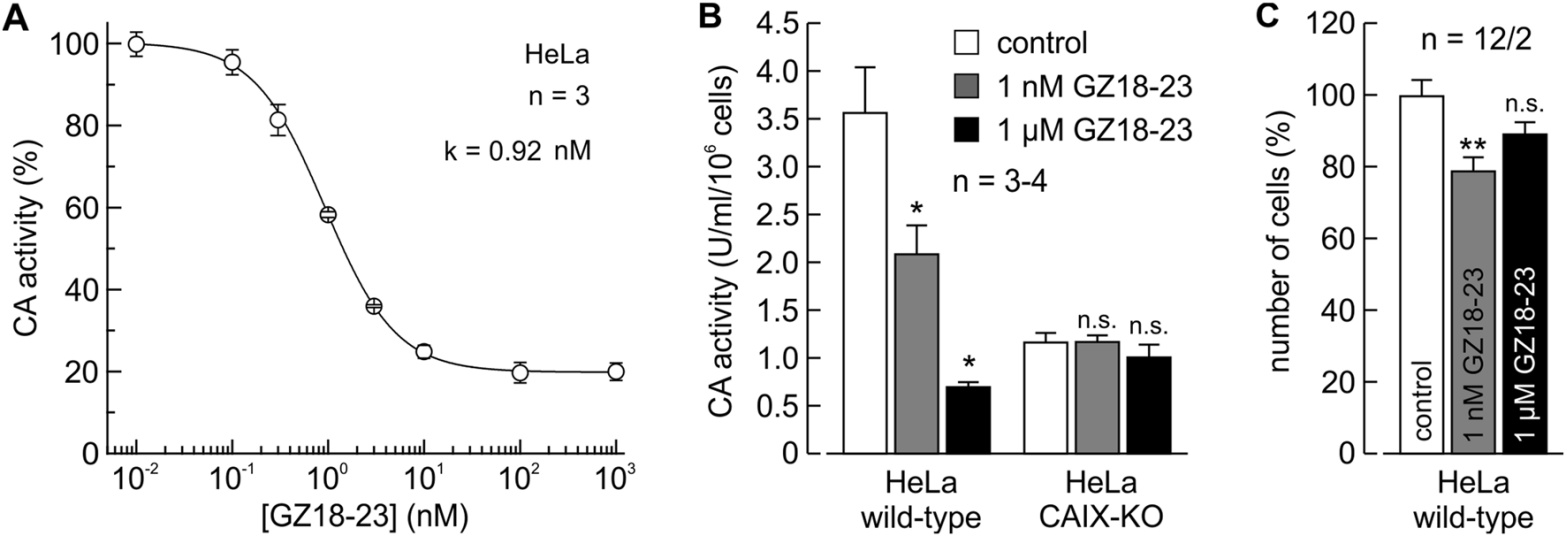
Inhibition of CA enzymatic activity by GZ18-23 in live hypoxic HeLa WT and CAIX-KO cell cultures by gas-analysis mass spectrometry. (**A**) Inhibition of CA activity by the inhibitor GZ18-23 in hypoxic HeLa cells. (**B**) Comparison of CA activity by gas-analysis MS of hypoxic HeLa WT and CAIX-KO cell cultures. (**C**) Viability of hypoxic HeLa cells upon application of the GZ18-23 inhibitor.

CAIX-KO was also validated by measuring the inhibition of CA activity in live cells by an inhibitor of CAIX, the GZ18-23. The compound showed strong inhibition of the CA activity by the gas-analysis mass spectrometry in hypoxic HeLa WT cells, but there was no effect in hypoxic HeLa CAIX-KO cells (Fig. 7).

The compound GZ18-23 was designed to retain the affinity towards CAIX of our previously discovered VD11-4-2. The new inhibitor had the same chemical structure as VD11-4–2 except that the hydroxyl group was replaced with the carboxylic group that improved its aqueous solubility and pharmacokinetic properties (Fig. 1). The inhibitor was highly effective to inhibit most of CA activity (Fig. 7A).

Under hypoxia, HeLa cells express numerous CA isozymes. Since the GZ18-23 inhibitor possesses high selectivity only for CAIX, the application of the inhibitor did not inhibit all CA activity. As seen in Fig. 7A, approximately 20% of overall CA activity remained uninhibited by the compound. This remaining activity was comparable to the CA activity in the HeLa CAIX-KO cells that have not been affected by the GZ18-23 inhibitor (Fig. 7B). The absence of CAIX in the HeLa CAIX-KO cells was possibly partially compensated by the increased overall activity of other CA isozymes as suggested by a slight increase of CA activity in the CAIX-KO cells as compared to the WT (Fig. 7B). The application of up to 1 µM concentration of GZ18-23 inhibited the CA activity but did not affect the viability of hypoxic HeLa cells (Fig. 7C).

### The CAIX-selective compound quantitative dosing profiles

After the confirmation of compound binding by microscopy, we attempted to determine the quantitative strength of interaction between the fluorescein-labeled compounds and CAIX expressed on cells. We have performed a series of experiments with various concentrations of the compounds applied to live cells grown in 12-well plates for 72 h and incubated for various time periods and oxygen tensions. These experiments yielded the quantitative affinities of the compounds in addition to the visualization by microscopy.

**Figure 8:**
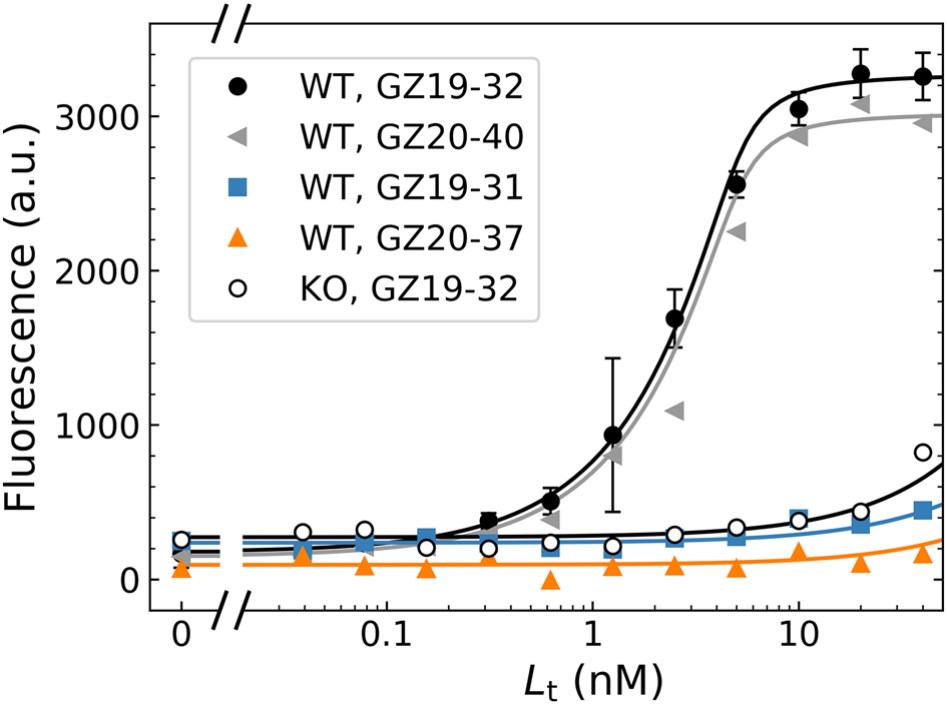
Quantitative dosing of the fluorescein-labeled compounds to HeLa cells (grown for 72 h under hypoxia). Dosing of GZ19-32 or GZ20-40 yielded similar dosing profiles (data points) with the lines fitted to the Morrison equation.

Application of the GZ19-32 compound at various concentrations yielded a concentration-dependent increase in fluorescence (Fig. 8). In the compound concentration range of 10 nM to 100 nM, the fluorescence increased only for the WT HeLa cells that have been grown under hypoxic conditions. The HeLa CAIX-KO cells grown under hypoxia did not show an increase in fluorescence of compounds bound to the cells, consistent with the microscopy observations (Fig. 3).

The *K*_d_ values for compounds GZ19-32 and GZ20-40 that are selective for CAIX were similar and equal to 0.15 nM (see below). The position of the curves did not indicate the *K*_d_ but instead showed the concentration of the interacting protein (CAIX) equal to approximately 5 nM in this figure, as obtained by the fit to Morrison equation. This value depends on the time used to grow the HeLa cells under hypoxia.

Application of small concentrations of the fluorescently labeled ligand GZ19-32 caused all of it to bind to CAIX on HeLa’s cell surface. Saturation was reached at a concentration of approximately 10 nM. The cells bound only GZ19-32 and GZ20-40 that were specific for CAIX and differed only in the length of the linker, and both contained the same CAIX-specific head-group. However, the GZ19-31 ligand, which contained the head-group specific for CAI isozyme, did not bind to the cells under the same conditions. Similarly, the compound GZ20-37, where sulfonamide amino group was substituted with the methyl group, did not bind CAIX in this concentration region. The binding was also abolished for the CAIX-KO HeLa cells (Fig. 8).

**Figure 9:**
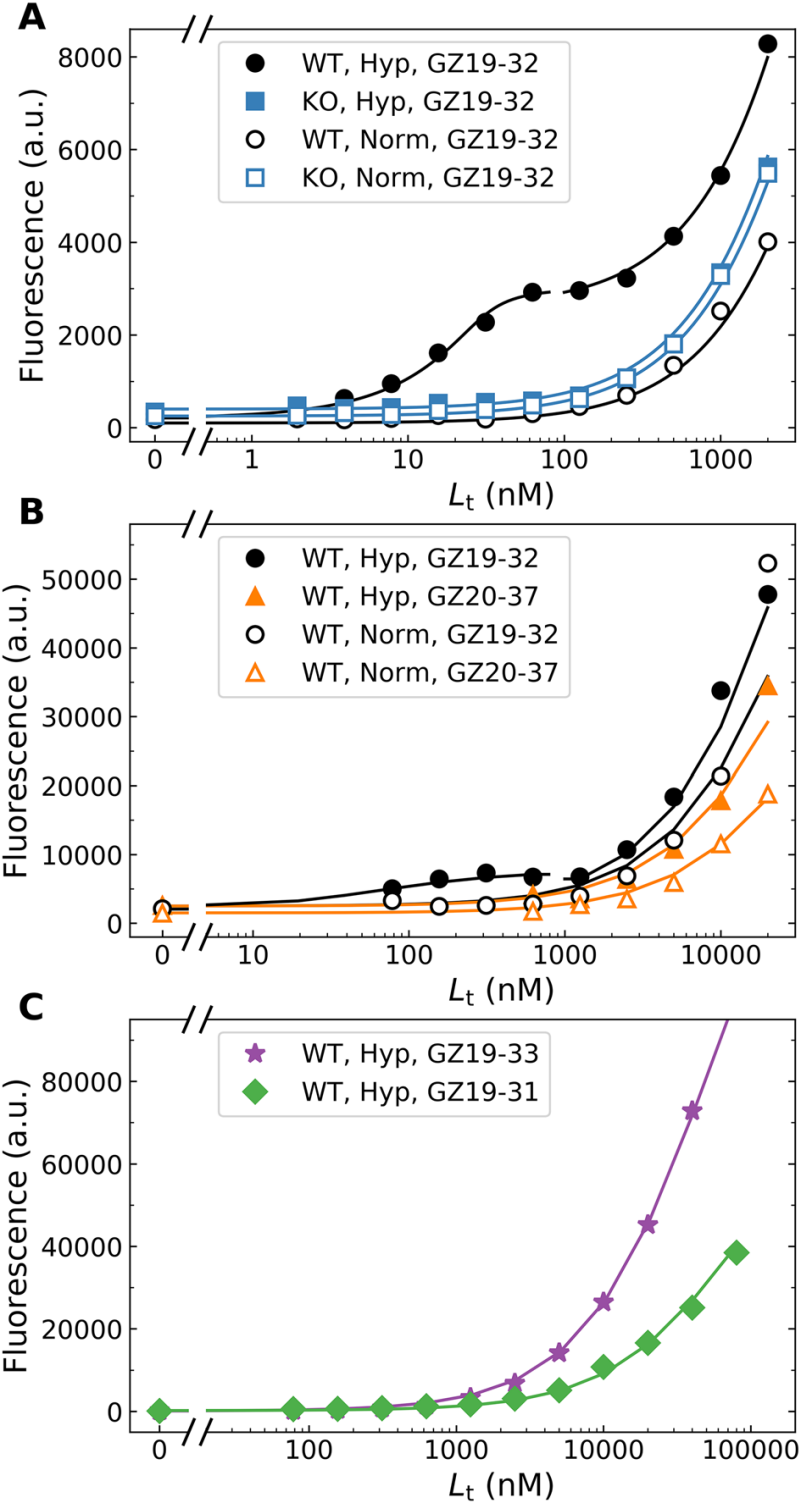
Dosing profiles of the fluorescein-labeled GZ compounds showing the specific binding of GZ19-32 to CAIX expressed only on hypoxia-grown WT HeLa cells (occurring at 100 nM after 5 min incubation (**A**) and at 500 nM after 2 min incubation (**B**) and the non-specific binding to unknown targets other than CAIX by all other compounds to both the WT and CAIX-KO HeLa cells grown both under hypoxia and normoxia conditions. (**C**) Non-specific binding of sulfonamide (GZ19-31) and non-sulfonamide methylated (GZ19-33) compound after 20 min incubation.

However, when micromolar concentrations were applied, all above listed compounds bound to all cells indicating that a low-affinity or non-specific binding occurred in the micromolar concentration range (Fig. 9). At concentrations exceeding approximately 100 nM, GZ19-32 began to bind also to the normoxia-grown WT HeLa cells and the CAIX-KO HeLa cells (Fig. 9A). Furthermore, the GZ20-37 methylated compound without the sulfonamide group (Fig. 9B) and GZ19-31 and GZ19-33 (Fig. 9C) also bound cells in the micromolar concentration range. All these results indicate that the compounds showed non-specific binding to sites other than the CAIX protein on the HeLa cell membrane above a certain concentration threshold (approximately 200 nM to 2 µM, depending on the incubation time).

**Figure 10:**
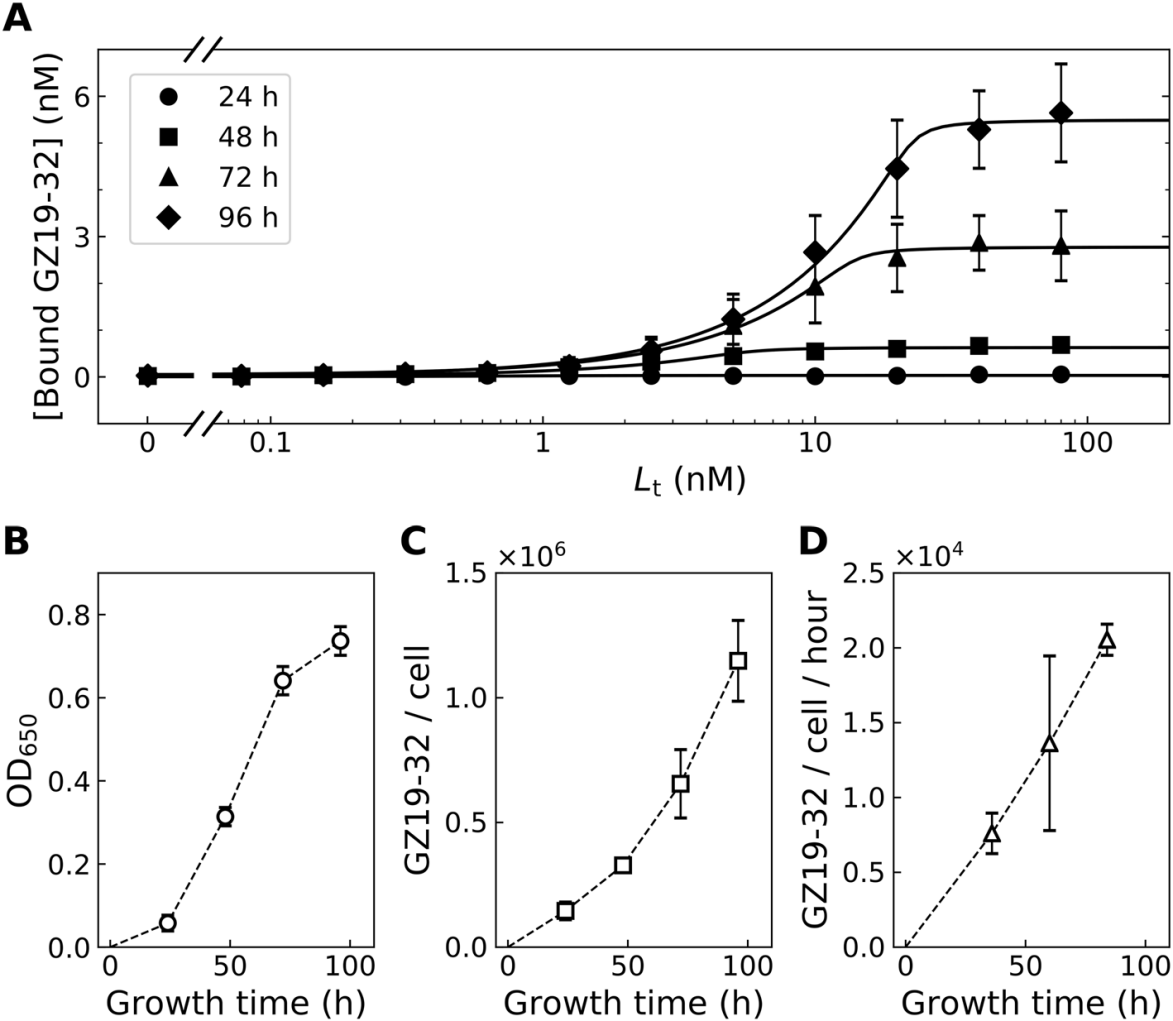
(**A**) The compound dosing profiles of GZ19-32 after growing HeLa cells for 24 (filled circles), 48 (filled squares), 72 (filled triangles), and 96 (filled diamonds) hours under hypoxia. The abscissa axis shows the concentration of added total GZ19-32 to the solution, while the ordinate shows the concentration part of the bound GZ19-32 compound as calculated from the fluorescence. (**B**) The rate of HeLa cell growth by following the optical density at 650 nm. (**C**) The number of bound GZ19-32 per HeLa cell. (**D**) The rate of CAIX production by each HeLa cell per hour based on data in panel (**C**).

The GZ19-32 compound dosing profiles were also determined for the WT HeLa cells grown under hypoxia for 1 to 4 days (Fig. 10). There was no binding observed after 24 h, but after 48 h binding occurred indicating that a detectable amount of CAIX protein has been produced. Substantial further increase in binding was observed after 72 and 96 h. Since the cells kept growing for 96 h (Fig. 10B), the amount of bound GZ19-32 was normalized by the number of cells determined by the cell counting resulting in the number of bound GZ19-32 molecules per cell (Fig. 10C). At approximately 80 h of growth under hypoxia, the number of bound GZ19-32 molecules per HeLa cell exceeded 1 million. Thus under assumption that the number of bound GZ19-32 represents the number of CAIX molecules, there were about a million CAIX molecules produced on average by each HeLa cell. The rate of CAIX production as followed by the number of bound GZ19-23 molecules reached around 20 thousand CAIX molecules produced by each cell per hour (Fig. 10D).

Figure 10A shows the bound concentration of GZ19-32 as a function of added compound concentration. Thus the plot can be used to double-check if the fitted curve position on the abscissa matches the saturation position on the ordinate. The Morrison fits, according to the abscissa, yielded the CAIX concentration of 0.9 nM at 24 h, 5.4 nM at 48 h, 14 nM at 72 h and 23 nM at 96 h. However, the saturation values on the ordinate were 0.03 nM at 24 h, 0.6 nM at 48 h, 2.8 nM at 72 h, and 5.5 nM at 96 h. Thus there was a significant mismatch between the two approaches to estimate the concentration of CAIX. The precision of Morrison fit is approximately plus minus two fold and the saturation values depend on the precision of fluorescence calibration. The mismatch appeared to be technique-dependent, and thus the ranking seems to be correct. The position of the curves on the abscissa did not indicate the affinity as is often assumed in similar dosing experiments, but instead it showed the concentration of CAIX. We may estimate that the CAIX concentration was approximately 0.3 nM at 24 h, 2 nM at 48 h, 6 nM at 72 h, and 12 nM at 96 h.

The above compound dosing profiles showed that in our cell culture conditions approximately 10 nM was necessary to saturate the CAIX produced by the cells. The compounds had an affinity *K*_d_ of 0.1 nM, but since the calculated concentration of CAIX was 10 nM, it would be necessary to add 10 nM to achieve saturation and full inhibition of the CAIX enzymatic activity (Figs. 8 and 10). Thus, the *K*_d_ of 0.1 nM cannot be directly determined by such dosing plots.

### Determination of non-fluorescent CA inhibitor affinity for cell-expressed CAIX by applying the competitive binding model

The next goal was to determine the affinity *K*_d_ of any CAIX inhibitor by incubating it in a mixture with the fluorescein-labeled compound. Any non-fluorescent compound can be applied by varying its concentration while keeping constant concentration of the fluorescent compound. When such a mixture of labeled and unlabeled compound is applied to cells, the fluorescein-labeled compound will compete with the unlabeled one and the binding profile will depend on the affinity and concentration of the unlabeled compound.

**Figure 11:**
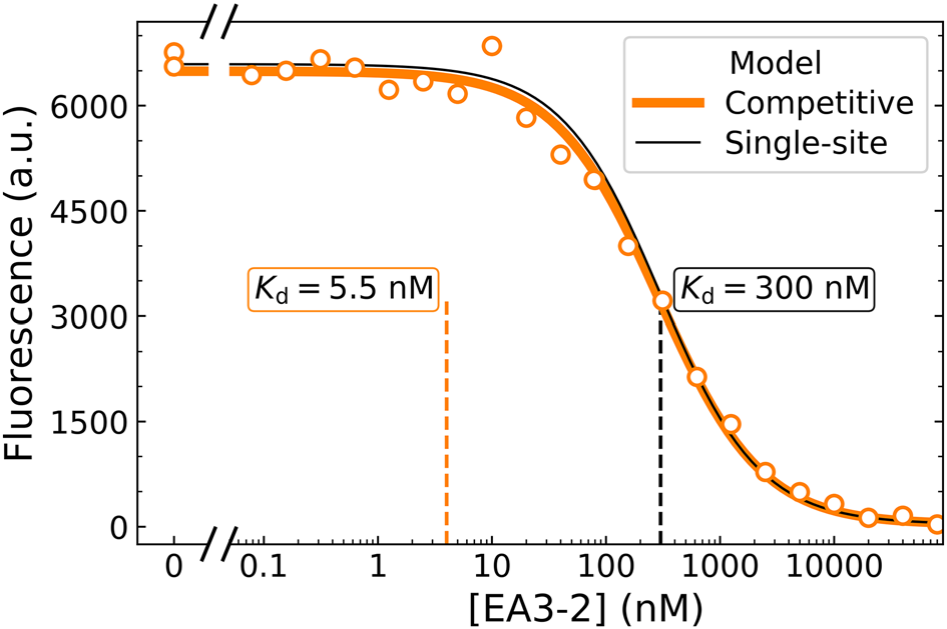
Determination of the binding constant of EA3-2 to CAIX expressed on live HeLa cells by competition with the fluorescein-containing GZ19-32. The concentration of GZ19-32 was constant at 10 nM while EA3-2 was dosed in two 12-step two-fold dilution series, first starting from 80 µM and the second – from 80 nM. The compounds GZ19-32 and EA3-2 were added together as a mixture. The single-site binding model would yield an uncorrected *K*_d_ = 300 nM, while the competitive model yielded a corrected *K*_d_ = 5.5 nM. Both models appear to fit the data well, but only the competitive model properly describes the situation.

In the competition experiment, the concentration of the fluorescently labeled GZ19-32 ligand was kept constant at 10 nM in a series of tubes while the concentration of the unlabeled compound (for example, EA3-2, shown in Fig. 11) was varied by performing serial twofold dilutions. Both the fluorescein-labeled and unlabeled compounds were applied simultaneously as a mixture to HeLa cells grown under hypoxia in 12-well plates. After 20 min incubation, unbound ligands were removed by extensive wash and the remaining cell fluorescence was measured. As seen in Fig. 11, at low concentrations EA3-2 was unable to out-compete the fluorescent GZ19-32 and thus the fluorescence was high. The value decreased in a dose-dependent manner upon increasing concentration of EA3-2.

Looking at the binding curve, it may seem that the expected *K*_d_ value of EA3-2 should be positioned near the concentration corresponding to 50% saturation, thus approximately 300 nM (Fig. 11). A single-site Morrison model determined such value (solid black line). However, this approach is incomplete because one must account for 10 nM of the competing fluorescent compound GZ19-32, which competes for the same binding site. Only after the application of the competitive binding model that accounted for all components, we obtained the corrected *K*_d_ (*K*_d_A—the affinity of the component A in the model) of EA3-2 equal to 5.5 nM. This value matched the *K*_d_ observed for EA3-2 binding to recombinant CAIX (TSA *K*_d_ = 4.0 nM, Table 2). Thus, there was an approximately 100-fold difference in the *K*_d_ result between the single-site model and the competitive model. Both models visually appeared to be perfectly matching the data-points. However, only the competitive model yielded the correct *K*_d_ values.

**Table 2:** Comparison between the *K*_d_ values for compound binding to recombinantly purified CAIX obtained by TSA and the CAIX expressed on the live cell surface obtained by competitive dosing.

Compound	*K*_d_ for purified CAIX by TSA, nM	Parameters of the fit in the competitive binding model			
		*K*_d_A, nM	*K*_d_B, nM	*L*_t_B, nM	*P*_t_, nM
**HeLa cells**					
AZM	21	15	0.2	10	2.0
SLC-0111	100	50	0.2	10	2.0
VD10-13	32	25	0.2	10	2.0
VD10-35	41	40	0.2	10	2.0
GZ18-23	0.033	0.30	0.2	10	2.0
VD11-4–2	0.032	0.07–0.30	0.2	10	2.0
EA3-2	4.0	5.5	0.2	10	2.0
**A549 cells**					
AZM	21	40	0.2	10	2.0
SLC-0111	100	300	0.2	10	2.0
VD10-13	32	32	0.2	10	2.0
VD10-35	41	40	0.2	10	2.0
GZ18-23	0.033	0.22	0.2	10	2.0
VD11-4–2	0.032	0.26–0.4	0.2	10	2.0
EA3-2	4.0	5	0.2	10	2.0

**Figure 12:**
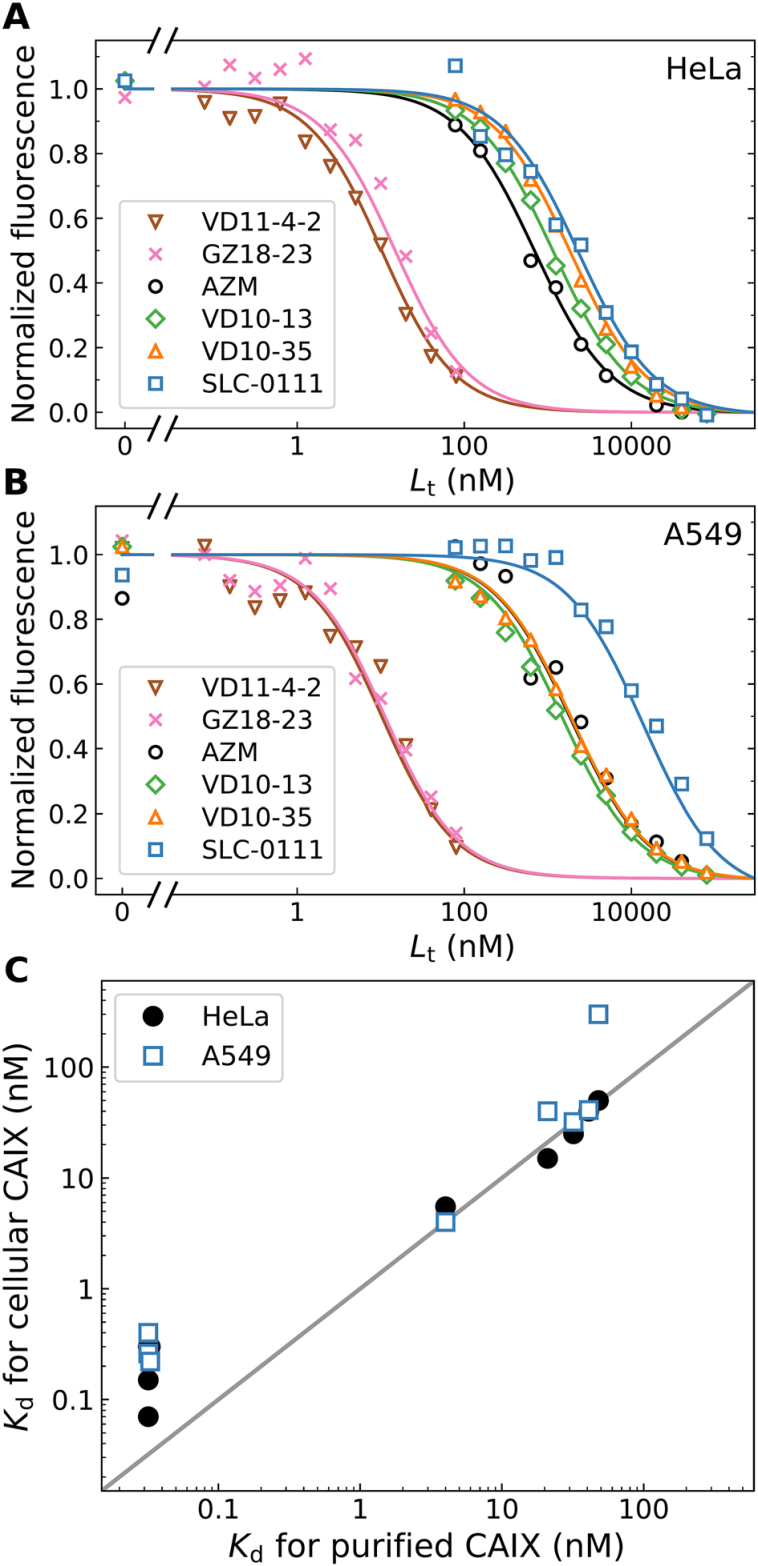
Competitive dosing of several CA inhibitors to HeLa (**A**) and A549 (**B**) cells grown under hypoxia in the presence of constant 10 nM concentration of fluorescein-labeled GZ19-32. (**C**) Comparison of *K*_d_ values for compound binding to CAIX expressed on live cell surface with the values obtained for compound binding to the purified CAIX by FTSA. Two cancer cell lines were tested—HeLa (filled circles) and A549 (open blue squares). The solid diagonal line shows a would-be perfect match between the two approaches to determine compound affinities for CAIX.

Parameters were obtained by applying the competitive binding model to the dosing data in Fig. 12.

Regression of the model parameters to the dosing data showed that the concentration of CAIX in the cell system was 2.0 nM, the competing fluorescent compound GZ19-32 (component B) bound with the *K*_d_B = 0.2 nM, while the compound of interest EA3-2 (component A) bound with the *K*_d_A = 5.5 nM. The 22-point EA3-2 compound dilution was prepared by two 12-step serial dilutions where the first started from 80 µM and the second started from 80 nM in twofold steps. The 11th point of the 80 µM series coincided with the first point of the 80 nM series and there were two control points at 0 nM from each dilution. Such 22-concentration dilutions covered an entire region from micromolar to sub-nanomolar and yielded the most accurate *K*_d_ values (Table 2).

A series of compounds were competitively dosed in a mixture with the constant concentration of GZ19-32 (10 nM) to determine their affinities for the cell-expressed CAIX. The competition curves showed well-defined profiles where the more potent binders out-competed GZ19-32 at lower concentrations than the weak ones (Fig. 12). The competitive dosing curves exhibited similar shapes but were shifted depending on the affinity of the studied compound. The affinities of the studied compounds were similar when dosing to HeLa and A549 cells grown under hypoxia that were included in this study to show that the effects apply not exclusively to the HeLa cells.

The tightest-binders of recombinant CAIX, namely, the inhibitors VD11-4–2 and GZ18-23, yielded the curves with the dosing midpoints around 10 nM. However, the application of the competition model yielded their true *K*_d_ values around 0.2 nM, which matched the values observed for compound binding to recombinantly purified CAIX as determined by TSA. The other four inhibitors exhibited much weaker affinity than the two most selective CAIX inhibitors. Acetazolamide, a widely used CA inhibitor, yielded the *K*_d_ of 15 nM, which was close to the value of 21 nM, obtained by TSA for its binding to recombinant CAIX. The SLC-0111 compound exhibited the *K*_d_ of 50 nM, close to the value of 100 nM, observed for its binding to recombinant CAIX (Table 2). The SLC-0111 appeared to be the weakest binder of cell-expressed CAIX among these tested inhibitors.

Most compounds bound with nearly identical affinities to both tested cell lines, HeLa and A549. However, there was a slight but reproducible mismatch for the SLC-0111 that bound A549 cells weaker than HeLa cells (experiment was repeated three times).

For the comparison, we performed VD11-4-2 competition experiment in hypoxic chamber without cell exposure to normoxia. The competitive curves were indistinguishable indicating that it was suitable to grow the cells for 72 h under hypoxia, while performing the competition experiment under normoxia for 1.5 h (Figure S4).

Figure 12C shows the match between the *K*_d_ values for compound binding to recombinant CAIX and CAIX on the cell surface. There was a relatively good agreement between the two methods that determines compound affinity for CAIX. However, the compounds VD11-4-2 and GZ18-23 had a lower affinity to the cell-expressed CAIX than to the purified CAIX. This difference was likely due to a rather long residence time, approximately 5 h for VD11-4-2^33,34^, and thus incomplete achievement of the equilibrium. However, an overall good match validated this approach to determine compound affinities for cell-expressed CAIX. We suggest that this method could also be used to determine affinities of any compounds for cellular CAIX that have not been previously tested for recombinant purified CAIX.

## Materials and methods

### Synthesis of fluorescein-labeled compounds

The GZ19-31 contains a *para*-substituted tetrafluorobenzenesulfonamide that is a marginal binder of CAIX, but selectively and with high-affinity recognizes CAI. The GZ19-32 is a VD11-4-2-based fluorescein-labeled compound that selectively and with high affinity binds to CAIX. The GZ20-37 is a methyl-substituted sulfonamide-containing compound lacking the amino group and thus unable to strongly bind CAIX, nor other CA isoforms. This compound is a mixture of 1:1 ratio of *para* and *meta*-substituted isomers. The GZ20-40 is analogous to GZ19-32, also designed to recognize CAIX, but contains a longer linker between the head-group and fluorescein. Compound GZ19-32 and GZ19-33 ^1^H and ^19^F NMR spectra are shown in figure S7.

#### *General procedure for the syntheses of GZ18-21*, *GZ19-28*

1,2,3,4,5-Pentafluorobenzenesulfonamide^13^ or 1,2,3,4,5-pentafluoro-6-(methylsulfonyl)benzene^35^(4.7 mmol, 1 eq.), triethylamine (1.67 mL, 12 mmol, 2.6 eq.) and 3-mercaptopropanoic acid (0.73 mL, 5.6 mmol, 1.2 eq.) were added to methanol (50 mL). The reaction was refluxed for 13 h, then evaporated under reduced pressure. The resultant precipitate was washed with water and purified by recrystallization.

***3-((2,3,5,6-tetrafluoro-4-sulfamoylphenyl)thio)propanoic acid (GZ18-21).***

Recrystallization was accomplished from water. Yield: 1.41 g, 83%; mp: 167–168 °C (lit.^13^ 168–169 °C).

***3-((2,3,5,6-tetrafluoro-4-(methylsulfonyl)phenyl)thio)propanoic acid (GZ19-28).***

Recrystallization was accomplished from methanol. Yield: 1.32 g, 84%; mp: 173–174 °C; TLC (EtOAc:CHCl_3_:AcOH, 45:45:1 v/v/v): *R*_f_ = 0.4; ^1^H NMR (400 MHz, DMSO-d_6_): δ 2.59 (2H, t, *J* = 6.8 Hz, C*H*_*2*_COOH), 3.24 (2H, t, *J* = 6.9 Hz, SC*H*_*2*_), 3.52 (3H, s, C*H*_*3*_SO_2_), 12.51 (1H, br. s, COO*H*); ^13^C NMR (100 MHz, DMSO-d_6_): δ 29.73 (t, *J*(^19^F–^13^C) = 3.3 Hz, S*C*H_2_), 35.06 (*C*H_2_COOH), 45.96 (*C*H_3_SO_2_), 119.45 (*C1*, t, *J*(^19^F–^13^C) = 15.1 Hz), 121.26 (*C4*, t, *J*(^19^F–^13^C) = 20.2 Hz), 143.97 (*C2* and *C6*, ddt, ^1^* J*(^19^F–^13^C) = 258.1 Hz, ^2^* J*(^19^F–^13^C) = 17.1 Hz, ^3^* J*(^19^F–^13^C) = 4.5 Hz), 146.93 (*C3* and *C5*, dm, *J*(^19^F–^13^C) = 245.7 Hz), 172.80 (*C*OOH); ^19^F NMR (376 MHz, DMSO-d_6_): δ -132.51 ÷ − 132.67 (2*F*, m), -138.65 ÷ − 138.81 (2*F*, m). HRMS (*m/z*): [M]^−^ calcd. for C_10_H_7_F_4_O_4_S_2_, 330.9727; found, 330.9730.

***3-((2,3,5,6-tetrafluoro-4-sulfamoylphenyl)sulfonyl)propanoic acid (GZ18-22)***^36^.

Appropriate compound (GZ18-21) (1.3 g, 3.6 mmol) was added to acetic acid (40 mL) and the mixture was heated for 13 h at 65–70 °C. H_2_O_2_ was added by portions of 0.1 mL every 15 min (overall used amount 3.7 mL of 40% H_2_O_2_). The progress of reaction was monitored by TLC. The solvent was then evaporated under reduced pressure and crude product was purified by recrystallization from water. Yield: 0.97 g, 69%; mp: 217–218 °C; TLC (EtOAc): *R*_f_ = 0.8; ^1^H NMR (400 MHz, DMSO-d_6_): δ 2.76 (2H, t, *J* = 7.0 Hz, C*H*_*2*_COOH), 3.81 (2H, t, *J* = 7.0 Hz, SO_2_C*H*_*2*_), 8.67 (2H, s, N*H*_*2*_SO_2_), 12.67 (1H, br. s, COO*H*); ^13^C NMR (100 MHz, DMSO-d_6_): δ 27.65 (*C*H_2_COOH), 53.19 (SO_2_*C*H_2_), 120.87 (*C1*, t, *J*(^19^F–^13^C) = 14.5 Hz), 128.26 (*C4*, t, *J*(^19^F–^13^C) = 15.1 Hz), 143.36 (*C2* and *C6*, dm, *J*(^19^F–^13^C) = 251.2 Hz), 145.10 (*C3* and *C5*, ddm, ^1^* J*(^19^F–^13^C) = 255.9 Hz, ^2^* J*(^19^F—^13^C) = 16.6 Hz), 171.55 (*C*OOH); ^19^F NMR (376 MHz, DMSO-d_6_): δ -135.89 (2*F*, dm, *J* = 12.4 Hz), -136.75 (2*F*, dm, *J* = 11.9 Hz).

***3-((2,3,5,6-tetrafluoro-4-(methylsulfonyl)phenyl)sulfonyl)propanoic acid (GZ19-30).***

Appropriate compound (GZ19-28) (0.13 g, 0.41 mmol) was added to acetic acid (10 mL) and the mixture was heated for 13 h at 90 °C. H_2_O_2_ was added by portions of 0.1 mL every 15 min (overall used amount 0.8 mL). The progress of reaction was monitored by TLC. The solvent was then evaporated under reduced pressure and crude product was purified by recrystallization from methanol and water (4:1 v/v). Yield: 0.96 g, 67%; mp: 230–231 °C; TLC (EtOAc:CHCl_3_:AcOH, 45:45:1 v/v/v): *R*_f_ = 0.51; ^1^H NMR (400 MHz, DMSO-d_6_): δ 2.76 (2H, t, *J* = 7.2 Hz, C*H*_*2*_COOH), 3.57 (3H, s, C*H*_*3*_SO_2_), 3.83 (2H, t, *J* = 7.1 Hz, SO_2_C*H*_*2*_), 12.68 (1H, br. s, COO*H*); ^13^C NMR (100 MHz, DMSO-d_6_): δ 27.66 (*C*H_2_COOH), 45.89 (*C*H_3_SO_2_), 53.21 (SO_2_*C*H_2_), 122.47 (*C1*, t, *J*(^19^F–^13^C) = 14.8 Hz), 124.76 (*C4*, t, *J*(^19^F–^13^C) = 15.0 Hz), 144.90 (*C2*, *C3*, *C5*, *C6*, dm, ^1^* J*(^19^F–^13^C) = 257.0 Hz), 171.47 (*C*OOH); ^19^F NMR (376 MHz, DMSO-d_6_): δ -135.32 (2*F*, dd, ^1^* J* = 26.5 Hz, ^2^* J* = 11.6 Hz), − 135.77 (2*F*, dd, ^1^* J* = 27.2 Hz, ^2^* J* = 11.7 Hz). HRMS (*m/z*): [M]^−^ calcd. for C_10_H_8_F_4_O_6_S_2_, 362.9620; found, 362.9622.

#### *General procedure for the syntheses of GZ18-23*, *GZ19-36*

Appropriate compound (GZ18-22, GZ19-30) (0.55 mmol, 1 eq.) was dissolved in DMSO (1.7 mL), then cyclooctylamine (0.15 mL, 1.1 mmol, 2 eq.) was added, and the reaction mixture was stirred for 5 h at room temperature, under argon atmosphere. Reaction progress was monitored using TLC. The reaction mixture was quenched with saturated sodium chloride solution. The resulting precipitate was extracted with ethyl acetate and dried over sodium sulfate. The extract was then evaporated under reduced pressure and purified with column chromatography.

***3-((2-(cyclooctylamino)-3,5,6-trifluoro-4-sulfamoylphenyl)sulfonyl)propanoic acid (GZ18-23*)**^36^.

Purified with column chromatography (EtOAc:CHCl_3_:AcOH, 45:45:1 v/v/v). Yield: 0.17 g, 66%; mp: 143–144 °C; TLC (EtOAc:CHCl_3_:AcOH, 45:45:1 v/v/v): *R*_f_ = 0.61; ^1^H NMR (400 MHz, DMSO-d_6_): δ 1.47–1.59 (12H, m, cyclooctane), 1.8–1.89 (2H, m, cyclooctane), 2.72 (2H, t, *J* = 6.9 Hz, C*H*_*2*_COOH), 3.75 (2H, t, *J* = 6.9 Hz, SO_2_C*H*_*2*_), 3.79 (1H, m, C*H* cyclooctane), 6.64 (1H, d, *J* = 8.5 Hz, N*H* cyclooctylamine), 8.36 (2H, s, N*H*_*2*_SO_2_), 12.03 (1H, s, COO*H*); ^13^C NMR (100 MHz, DMSO-d_6_): δ 23.27 (cyclooctane), 25.45 (cyclooctane), 27.19 (cyclooctane), 27.67 (*C*H_2_COO), 32.65 (cyclooctane), 52.90 (SO_2_*C*H_2_), 55.87 (*C*H cyclooctane, d, *J*(^19^F–^13^C) = 11.3 Hz), 114.75 (*C1*, dd, ^1^* J*(^19^F–^13^C) = 12.8 Hz, ^2^* J*(^19^F–^13^C) = 5.5 Hz), 128.08 (*C4*, dd, ^1^* J*(^19^F–^13^C) = 18.2 Hz, ^2^* J*(^19^F–^13^C) = 13.9 Hz), 135.34 (*C2*, dd, ^1^* J*(^19^F–^13^C) = 14.6 Hz, ^2^* J*(^19^F–^13^C) = 1.6 Hz), 137.25 (*C5*, ddd, ^1^* J*(^19^F–^13^C) = 245.7 Hz, ^2^* J*(^19^F–^13^C) = 17.5 Hz, ^3^* J*(^19^F–^13^C) = 5.0 Hz), 144.43 (*C3*, d, *J*(^19^F–^13^C) = 253.5 Hz), 146.28 (C6, ddd, ^1^* J*(^19^F–^13^C) = 250.5 Hz, ^2^* J*(^19^F–^13^C) = 16.8 Hz, ^3^* J*(^19^F–^13^C) = 3.9 Hz), 171.52 (*C*OOH); ^19^F NMR (376 MHz, DMSO-d_6_): δ -124.87-(-125.02) (C3**-***F*, m), -133.76 (C5**-***F*, dd, ^1^* J* = 27.3 Hz, ^2^* J* = 12.4 Hz ), − 150.55 (C6**-***F*, dd, ^1^* J* = 26.8 Hz, ^2^* J* = 6.5 Hz).

***3-((2-(cyclooctylamino)-3,5,6-trifluoro-4-(methylsulfonyl)phenyl)sulfonyl)propanoic acid and 3-((3-(cyclooctylamino)-2,5,6-trifluoro-4-(methylsulfonyl)phenyl)sulfonyl)propanoic acid (GZ19-36).***

*-Meta* (3-((3-(cyclooctylamino)-2,5,6-trifluoro-4-(methylsulfonyl)phenyl)sulfonyl)propanoic acid (GZ19-36)) and *–orto* (3-((2-(cyclooctylamino)-3,5,6-trifluoro-4-(methylsulfonyl)phenyl)sulfonyl)propanoic acid) isomers were not separated and obtained in 9:11 ratio (unspecified isomers). Purified with column chromatography (EtOAc:CHCl_3_:AcOH, 45:45:1 v/v/v). Yield: 0.11 g, 47%; TLC (EtOAc:CHCl_3_:AcOH, 45:45:1 v/v/v): *R*_f_ = 0.36; ^1^H NMR (400 MHz, DMSO-d_6_): δ *isomer 1* (45%)–1.47–1.69 (12H, m, cyclooctane), 1.81–1.91 (2H, m, cyclooctane), 2.75 (2H, t, *J* = 7.0 Hz, C*H*_*2*_COOH), 3.49 (3H, s, C*H*_*3*_SO_2_), 3.77 (2H, t, *J* = 6.9 Hz, SO_2_C*H*_*2*_), 3.81 (1H, m, C*H* cyclooctane), 6.67 (1H, d, *J* = 8.8 Hz, N*H* cyclooctylamine), 12.71 (1H, s, COO*H*), *isomer 2* (55%)–1.47–1.69 (12H, m, cyclooctane), 1.81–1.91 (2H, m, cyclooctane), 2.72 (2H, t, *J* = 6.9 Hz, C*H*_*2*_COOH), 3.51 (3H, s, C*H*_*3*_SO_2_), 3.75 (2H, t, *J* = 6.9 Hz, SO_2_C*H*_*2*_), 3.81 (1H, m, C*H* cyclooctane), 6.71 (1H, d, *J* = 8.7 Hz, N*H* cyclooctylamine), 12.71 (1H, s, COO*H*); ^13^C NMR (100 MHz, DMSO-d_6_): δ 23.22; 23.25; 25.46 (2), 27.21; 27.25; 27.68; 27.70; 32.62; 32.65; 45.87; 45.93; 52.94 (2), 55.91 (d, *J* = 11.3 Hz), 56.06 (d, *J* = 10.9 Hz), 115.99 (dd, ^1^* J*(^19^F–^13^C) = 12.6 Hz, ^2^* J*(^19^F–^13^C) = 5.9 Hz (2)), 124.81 (dd, ^1^* J*(^19^F–^13^C) = 18.1 Hz, ^2^* J*(^19^F–^13^C) = 3.2 Hz (2)), 134.98 (d, *J*(^19^F–^13^C) = 12.4 Hz), 135.61 (d, *J*(^19^F–^13^C) = 13.7 Hz), 137.99 (dm, *J*(^19^F—^13^C) = 248.3 Hz (2)), 145.41 (d, *J*(^19^F—^13^C) = 254.2 Hz (2)), 146.34 (dm, ^1^* J*(^19^F–^13^C) = 260.3 Hz, (2)), 171.48; 171.52; ^19^F NMR (376 MHz, DMSO-d_6_): δ *isomer 1* (45%)–− 123.61–(− 123.73) (C3-*F*, m), − 134.06 (C5**-***F*, dd, ^1^* J* = 27.2 Hz, ^2^* J* = 12.3 Hz), − 150.16 (C6**-***F*, dd, ^1^* J* = 26.5 Hz, ^2^* J* = 8.6 Hz), *isomer 2* (55%)–− 124.87-(-125.01) (C3-*F*, m), − 133.17 (C5**-***F*, dd, ^1^* J* = 27.2 Hz, ^2^* J* = 12.5 Hz ), -151.08 (C6**-***F*, dd, ^1^* J* = 27.2 Hz, ^2^* J* = 8.2 Hz). HRMS (*m/z*): [M]^+^ calcd. for C_18_H_24_F_3_NO_6_S_2_, 472.1070; found, 472.1071.

#### *General procedure for the syntheses of GZ19-31*, *GZ19-32*, *GZ19-33*, *GZ20-37, GZ20-40*

The mixture of appropriate starting material (GZ18-22, GZ18-23, GZ19-28, GZ19-36, GZ20-39) (0.23 mmol, 1.1 eq.), EDC (0.065 g, 0.42 mol, 2 eq.), 5-aminofluoresceine (0.052 g, 0.21 mmol, 1 eq.) and 5 mL of dry pyridine was stirred in room temperature under argon atmosphere for 24 h. Reaction progress was monitored using TLC. The solvent was evaporated under reduced pressure, the crude product was poured into saturated sodium chloride solution (5 mL), acidified till neutral pH and extracted with ethyl acetate. The organic layer was washed with sat. sodium chloride solution and dried over sodium sulfate. The solvent was evaporated under reduced pressure, and residue was purified with column chromatography.

***N-(3',6'-dihydroxy-3-oxo-3H-spiro[isobenzofuran-1,9'-xanthen]-5-yl)-3-((2,3,5,6-tetrafluoro-4-sulfamoylphenyl)sulfonyl)propanamide (GZ19-31).***

Purified with column chromatography (acetone:CHCl_3_, 1:1 v/v). Yield: 6.1 mg, 6%; mp: 175–176 °C; TLC (EtOAc:CHCl_3_:AcOH, 45:45:1 v/v/v): *R*_f_ = 0.26; ^1^H NMR (400 MHz, DMSO-d_6_): δ 2.97 (2H, t, *J* = 7.2 Hz, C*H*_*2*_CONH), 3.95 (2H, t,* J* = 7.2 Hz, SO_2_C*H*_*2*_), 6.54 (2H, dd, ^1^*J* = 8.7 Hz, ^2^*J* = 2.3 Hz, C2’’-*H* and C7’’-*H*), 6.59 (2H, d, *J* = 8.6 Hz, C1’’-*H* and C7’’-*H*), 6.67 (2H, d, *J* = 2.3 Hz, C4’’-*H* and C5’’-*H*), 7.22 (1H, d, *J* = 8.4 Hz, C7’-*H*), 7.74 (1H, dd, ^1^*J* = 8.4 Hz, ^2^*J* = 1.9 Hz, C6’-*H*), 8.22 (1H, d, *J* = 1.7 Hz, C4’-*H*), 8.66 (2H, s, N*H*_*2*_SO_2_), 10.13 (2H, s, O*H*), 10.64 (1H, s, N*H*CO); ^13^C NMR (100 MHz, DMSO-d_6_): δ 30.07 (*C*H_2_CONH), 53.05 (SO_2_*C*H_2_), 83.57 (quaternary *C*), 102.65 (xanthene), 110.12 (xanthene), 113.04 (xanthene), 113.85 (Isobenzofuranone), 120.74 (*C1*, t, *J*(^19^F–^13^C) = 15.2 Hz), 125.05 (Isobenzofuranone), 126.70 (Isobenzofuranone), 127.52 (Isobenzofuranone), 128.34 (*C4*, t, *J*(^19^F–^13^C) = 15.3 Hz), 129.60 (xanthene), 140.73 (Isobenzofuranone), 143.45 (*C2* and *C6*, dm, *J*(^19^F–^13^C) = 262.3 Hz), 145.01 (*C3* and *C5*, dm, *J*(^19^F–^13^C) = 265.5 Hz), 147.44 (Isobenzofuranone), 152.36 (xanthene), 159.93 (*C*–OH), 168.19 (*C*ON), 169.00 (*C*OO); ^19^F NMR (376 MHz, DMSO-d_6_): δ − 135.64 (2*F*, dm, *J* = 24.8 Hz), − 136.63 (2*F*, dm, *J* = 24.2 Hz). HRMS (*m/z*): [M]^−^ calcd. for C_29_H_18_F_4_N_2_O_10_S_2_, 693.0262; found, 693.0248.

***3-((2-(cyclooctylamino)-3,5,6-trifluoro-4-sulfamoylphenyl)sulfonyl)-N-(3',6'-dihydroxy-3-oxo-3H-spiro[isobenzofuran-1,9'-xanthen]-5-yl)propanamide (GZ19-32).***

Purified with column chromatography (acetone:CHCl_3_, 2:3 v/v). Yield: 52 mg, 30%; mp: 189–190 °C; TLC (acetone:CHCl_3_, 3:2 v/v): *R*_f_ = 0.54; ^1^H NMR (400 MHz, DMSO-d_6_): δ 1.36–1.70 (12H, m, cyclooctane), 1.76–1.89 (2H, m, cyclooctane), 2.94 (2H, t, *J* = 6.8 Hz, C*H*_*2*_CONH), 3.75 (1H, m, C*H* cyclooctane), 3.90 (2H, t, *J* = 6.8 Hz, SO_2_C*H*_*2*_), 6.50 (2H, dd, ^1^*J* = 8.8 Hz, ^2^*J* = 2.2 Hz, C2’’-*H* and C7’’-*H*), 6.58 (2H, d, *J* = 8.6 Hz, C1’’-*H* and C7’’-*H*), 6.64 (1H, d, *J* = 8.3 Hz, N*H* cyclooctylamine), 6.68 (2H, d, *J* = 2.1 Hz, C4’’-*H* and C5’’-*H*), 7.21 (1H, d, *J* = 8.4 Hz, C7’-*H*), 7.75 (1H, dd, ^1^* J* = 8.3 Hz, ^2^* J* = 1.8 Hz, C6’-*H*), 8.23 (1H, d, *J* = 1.6 Hz, C4’-*H*), 8.34 (2H, s, N*H*_*2*_SO_2_), 10.12 (2H, s, O*H*), 10.61 (1H, s, N*H*CO); ^13^C NMR (100 MHz, DMSO-d_6_): δ 23.25 (cyclooctane), 25.42 (cyclooctane), 27.17 (cyclooctane), 29.77 (*C*H_2_CONH), 32.73 (cyclooctane), 52.97 (SO_2_*C*H_2_), 55.90 (*C*H cyclooctane, d, *J*(^19^F–^13^C) = 10.9 Hz), 83.57 (quaternary *C*), 102.68 (xanthene), 110.14 (xanthene), 112.99 (xanthene), 113.87 (isobenzofuranone), 114.71 (*C1*, dd, ^1^* J*(^19^F–^13^C) = 12.6 Hz, ^2^* J*(^19^F–^13^C) = 6.4 Hz), 124.99 (isobenzofuranone), 126.71 (isobenzofuranone), 127.49 (isobenzofuranone), 127.99 (*C4*, dd, ^1^* J*(^19^F–^13^C) = 17.9 Hz, ^2^* J*(^19^F–^13^C) = 3.2 Hz), 129.51 (xanthene), 135.34 (*C2*, d, *J*(^19^F-^13^C) = 13.9 Hz), 137.31 (*C5*, dm, *J*(^19^F–^13^C) = 251.7 Hz), 140.79 (isobenzofuranone), 144.91 (*C3*, d, *J*(^19^F–^13^C) = 262.9 Hz), 146.27 (*C6*, ddm, ^1^* J*(^19^F–^13^C) = 255.1 Hz, ^2^*J*(^19^F–^13^C) = 18.6 Hz), 147.35 (isobenzofuranone), 152.38 (xanthene), 159.93 (*C*–OH), 168.14 (*C*OO), 169.01 (*C*ON); ^19^F NMR (376 MHz, DMSO-d_6_): δ − 125.05 (C3-*F*, dd, ^1^*J* = 12.6 Hz, ^2^*J* = 6.8 Hz), − 134.00 (C5**-***F*, dd, ^1^* J* = 27.4 Hz, ^2^*J* = 12.4 Hz), − 150.65 (C6**-***F*, dd, ^1^*J* = 27.0 Hz, ^2^*J* = 6.7 Hz).nHRMS (*m/z*): [M]^-^ calcd. for C_37_H_34_F_3_N_3_O_10_S_2_, 802.1710; found, 802.1711.

***N-(3',6'-dihydroxy-3-oxo-3H-spiro[isobenzofuran-1,9'-xanthen]-5-yl)-3-((2,3,5,6-tetrafluoro-4-(methylsulfonyl)phenyl)thio)propanamide (GZ19-33).***

Purified with column chromatography (acetone:CHCl_3_, 2:3 v/v). Yield: 59 mg, 47%; mp: 181–182 °C; TLC (EtOAc:CHCl_3_:AcOH, 45:45:1 v/v/v): *R*_f_ = 0.34; ^1^H NMR (400 MHz, DMSO-d_6_): δ 2.77 (2H, t, *J* = 6.5 Hz, C*H*_*2*_CONH), 3.40 (2H, t, *J* = 6.6 Hz, SC*H*_*2*_), 3.51 (3H, s, C*H*_*3*_SO_2_), 6.55 (2H, dd, ^1^*J* = 8.9 Hz, ^2^* J* = 2.1 Hz, C2’’-*H* and C7’’-*H*), 6.61 (2H, d, *J* = 8.7 Hz, C1’’-*H* and C7’’-*H*), 6.68 (2H, d, *J* = 2.0 Hz, C4’’-*H* and C5’’-*H*), 7.22 (1H, d, *J* = 8.4 Hz, C7’-*H*), 7.76 (1H, dd, ^1^*J* = 8.1 Hz, ^2^*J* = 1.3 Hz, C6’-*H*), 8.27 (1H, d, *J* = 1.2 Hz, C4’-*H*), 10.13 (2H, s, O*H*), 10.51 (1H, s, N*H*CO); ^13^C NMR (100 MHz, DMSO-d_6_): δ 30.07 (S*C*H_2_), 37.46 (*C*H_2_CON), 45.96 (*C*H_3_SO_2_), 83.54 (quaternary *C*), 102.66 (xanthene), 110.16 (xanthene), 113.03 (xanthene), 113.76 (isobenzofuranone), 119.54 (*C1*, t, *J*(^19^F–^13^C) = 15.0 Hz), 121.18 (*C4*, t, *J*(^19^F–^13^C) = 20.1 Hz), 124.98 (isobenzofuranone), 126.67 (isobenzofuranone), 127.47 (isobenzofuranone), 129.59 (xanthene), 140.82 (isobenzofuranone), 143.91 (*C2* and *C6*, ddt, ^1^*J*(^19^F–^13^C) = 257.9 Hz, ^2^*J*(^19^F—^13^C) = 17.3 Hz, ^3^* J*(^19^F–^13^C) = 4.6 Hz), 147.08 (*C3* and *C5*, ddm, ^1^*J*(^19^F–^13^C) = 245.4 Hz, ^2^* J*(^19^F–^13^C) = 14.7 Hz), 147.39 (isobenzofuranone), 152.37 (xanthene), 159.92 (*C*–OH), 169.04 (*C*OO), 169.82 (*C*ON); ^19^F NMR (376 MHz, DMSO-d_6_): δ -132.04 (2*F*, dm, *J* = 24.5 Hz), − 138.74 (2*F*, dm, *J* = 24.5 Hz). HRMS (*m/z*): [M]^−^ calcd. for C_30_H_19_F_4_NO_8_S_2_, 660.0410; found, 660.0408.

***3-((2-(cyclooctylamino)-3,5,6-trifluoro-4-(methylsulfonyl)phenyl)sulfonyl)-N-(3',6'-dihydroxy-3-oxo-3H-spiro[isobenzofuran-1,9'-xanthen]-5-yl)propanamide and 3-((3-(cyclooctylamino)-2,5,6-trifluoro-4-(methylsulfonyl)phenyl)sulfonyl)-N-(3',6'-dihydroxy-3-oxo-3H-spiro[isobenzofuran-1,9'-xanthen]-5-yl)propanamide (GZ20-37).***

Purified with column chromatography (acetone:CHCl_3_, 1:1 v/v). Yield: 30 mg, 23%; TLC (EtOAc:CHCl_3_:AcOH, 45:45:1 v/v/v): *R*_f_ = 0.8; ^1^H NMR (400 MHz, DMSO-d_6_): δ 1.39–1.68 (24H, m, cyclooctane), 1.78–1.88 (4H, m, cyclooctane), 2.95 (2H, t, *J* = 6.6 Hz, C*H*_*2*_CONH), 2.98 (2H, t, *J* = 6.6 Hz, C*H*_*2*_CONH), 3.45 (3H, s, C*H*_*3*_SO_2_), 3.48 (3H, s, C*H*_*3*_SO_2_), 3.75 (2H, m, C*H* cyclooctane), 3.92 (2H, t, *J* = 6.2 Hz, SO_2_C*H*_*2*_), 3.93 (2H, t, *J* = 6.2 Hz, SO_2_C*H*_*2*_), 6.54 (4H, br. dd, ^1^* J* = 8.5 Hz, ^2^* J* = 1.4 Hz, C2’’-*H* and C7’’-*H*), 6.57 (2H, d, *J* = 8.6 Hz, C1’’-*H* and C7’’-*H*), 6.58 (2H, d, *J* = 8.6 Hz, C1’’-*H* and C7’’-*H*), 6.62 (1H, d, *J* = 9.2 Hz, N*H* cyclooctylamine), 6.67 (4H, br. s, C4’’-*H* and C5’’-*H*), 6.70 (1H, d, *J* = 9.4 Hz, N*H* cyclooctylamine), 7.19 (1H, d, *J* = 7.5 Hz, C7’-*H*), 7.21 (1H, d, *J* = 7.8 Hz, C7’-*H*), 7.71 (1H, dd, ^1^* J* = 8.3 Hz, ^2^* J* = 1.8 Hz, C6’-*H*), 7.74 (1H, dd, ^1^* J* = 8.3 Hz, ^2^* J* = 1.8 Hz, C6’-*H*), 8.19 (1H, d, *J* = 1.5 Hz, C4’-*H*) ir 8.23 (1H, d, *J* = 1.5 Hz, C4’-*H*), 10.13 (4H, s, O*H*), 10.64 (2H, s, N*H*CO); ^13^C NMR (100 MHz, DMSO-d_6_): δ 23.16 (cyclooctane), 23.25 (cyclooctane), 25.38 (cyclooctane), 25.45 (cyclooctane), 27.15 (cyclooctane), 27.27 (cyclooctane), 29.86 (2C, *C*H_2_CONH), 32.55 (cyclooctane), 32.79 (cyclooctane), 45.84 (2C, *C*H_3_SO_2_), 52.72 (SO_2_*C*H_2_), 52.96 (SO_2_*C*H_2_), 55.90 (*C*H cyclooctane, d, *J*(^19^F–^13^C) = 11.1 Hz), 56.03 (*C*H cyclooctane, d, *J*(^19^F–^13^C) = 11.1 Hz), 83.50 (quaternary *C*), 83.52 (quaternary *C*), 102.66 (2C, xanthene), 110.14 (2C, xanthene), 112.99 (2C, xanthene), 113.76 (2C, isobenzofuranone), 115.05 (2C, *C1*), 124.97 (2C, isobenzofuranone), 126.62 (2C, isobenzofuranone), 127.49 (2C, isobenzofuranone), 128.17 (2C, *C4*), 129.49 (xanthene), 129.54 (xanthene), 134.97 (*C2*, d, *J*(^19^F-^13^C) = 15.1 Hz), 134.97 (*C2*, d, *J*(^19^F-^13^C) = 15.4 Hz), 137.65 (2C, *C5*, dm, *J*(^19^F–^13^C) = 267.0 Hz), 140.69 (isobenzofuranone), 140.77 (isobenzofuranone), 143.45 (*C3*, d, *J*(^19^F–^13^C) = 251.6 Hz), 146.17 (*C6*, dm, *J*(^19^F–^13^C) = 262.3 Hz), 147.38 (2C, isobenzofuranone), 152.37 (2C, xanthene), 159.93 (2C, *C*–OH), 168.11 (*C*OO), 168.15 (*C*OO), 168.92 (*C*ON), 168.96 (*C*ON); ^19^F NMR (376 MHz, DMSO-d_6_): δ -123.63 (C3-*F*, dd, ^1^*J* = 12.9 Hz, ^2^*J* = 8.9 Hz), -125.32 (C3-*F*, dd, ^1^*J* = 12.2 Hz, ^2^*J* = 8.9 Hz), -133.28 (C5**-***F*, dd, ^1^*J* = 26.5 Hz, ^2^*J* = 7.0 Hz ), -134.15 (C5**-***F*, dd, ^1^*J* = 27.2 Hz, ^2^*J* = 12.5 Hz ), -150.08 (C6**-***F*, dd, ^1^*J* = 27.2 Hz, ^2^*J* = 8.7 Hz), -151.23 (C6**-***F*, dd, ^1^*J* = 27.9 Hz, ^2^*J* = 8.3 Hz). HRMS (*m/z*): [M]^+^ calcd. for C_38_H_35_F_3_N_2_O_10_S_2_, 801.1763; found, 801.1756.

***Tert-butyl 4-(3-((2-(cyclooctylamino)-3,5,6-trifluoro-4 sulfamoylphenyl)sulfonyl)propanamido) butanoate (GZ20-38)***.

The mixture of GZ18-23 (250 mg, 0.53 mmol, 1 eq.), EDC (246.5 mg, 1.59 mmol, 3 eq.), HOBT (107.2 mg, 0.80 mmol, 1.5 eq.), *tert*-butyl 4-aminobutanoate (155.5 mg, 0.80 mmol, 1.5 eq.) and 12 mL of dry pyridine was stirred in room temperature under argon atmosphere for 24 h. Reaction progress was monitored using TLC (CHCl_3_:acetone, 4:1 v/v): *R*_f_ = 0.4. Solvent was evaporated under reduced pressure, crude product was poured into saturated sodium chloride solution (10 mL), acidified till neutral pH and extracted with ethyl acetate. Organic layer was washed with sat. sodium cloride solution and dried over sodium sulfate. Solvent was evaporated under reduced pressure and residue was purified with column chromatography (same eluent as for TLC). Yield: 0.18 g, 55%; mp: 125–126 °C; ^1^H NMR (400 MHz, DMSO-d_6_): δ 1.39 (9H, s, *tert*-Bu), 1.43–1.70 (14H, m, cyclooctane, NHCH_2_C*H*_*2*_), 1.8–1.89 (2H, m, cyclooctane), 2.19 (2H, t, *J* = 7.5 Hz, C*H*_*2*_COO), 2.57 (2H, t, *J* = 7.1 Hz, C*H*_*2*_CON), 2.97 (2H, q, *J* = 6.6 Hz, NHC*H*_*2*_), 3.74 (2H, t, *J* = 7.2 Hz, SO_2_C*H*_*2*_), 3.79 (1H, m, C*H* cyclooctane), 6.6 (1H, d, *J* = 8.4 Hz, N*H* cyclooctylamine), 8.05 (1H, t, *J* = 5.5 Hz, N*H*CO), 8.36 (2H, s, N*H*_*2*_SO_2_); ^13^C NMR (100 MHz, DMSO-d_6_): δ 23.27 (cyclooctane), 24.91 (NHCH_2_*C*H_2_), 25.46 (cyclooctane), 27.21 (cyclooctane), 28.21 ((*C*H_3_)_3_C), 28.29 (*C*H_2_CONH), 32.61 (*C*H_2_COO), 32.68 (cyclooctane), 38.54 (CONH*C*H_2_), 53.12 (SO_2_*C*H_2_), 55.87 (d, *J*(^19^F–^13^C) = 11.4 Hz, *C*H cyclooctane), 79.99 ((CH_3_)_3_*C*), 114.85 (*C1*, dd, ^1^* J*(^19^F–^13^C) = 12.4 Hz, ^2^* J*(^19^F–^13^C) = 5.8 Hz), 128.01 (*C4*, dd, ^1^* J*(^19^F–^13^C) = 18.4 Hz, ^2^* J*(^19^F–^13^C) = 14.1 Hz), 135.29 (*C2*, dm, *J*(^19^F–^13^C) = 13.7 Hz), 137.12 (*C5*, dm, *J*(^19^F–^13^C) = 240.2 Hz), 144.46 (*C3*, dm, *J*(^19^F–^13^C) = 252.9 Hz), 146.22 (C6, ddd, ^1^* J*(^19^F–^13^C) = 250.1 Hz, ^2^* J*(^19^F–^13^C) = 16.1 Hz, ^3^* J*(^19^F–^13^C) = 5.9 Hz), 168.20 (*C*ON), 172.41 (*C*OO); ^19^F NMR (376 MHz, DMSO-d_6_): δ -125.04 (C3**-***F*, s), -133.93 (C5**-***F*, dd, ^1^*J* = 27.0 Hz, ^2^*J* = 12.5 Hz ), -150.61 (C6**-***F*, dd, ^1^* J* = 27.1 Hz, ^2^*J* = 6.7 Hz). HRMS (*m/z*): [M]^+^ calcd. for C_25_H_39_F_3_N_3_O_7_S_2_, 614.2176; found, 614.2170.

***4-(3-((2-(cyclooctylamino)-3,5,6-trifluoro-4-sulfamoylphenyl)sulfonyl)propanamido)butanoic acid (GZ20-39).***

The mixture of GZ20-38 (50 mg, 0.051 mmol, 1 eq.), TFA (1 mL, 13.07 mmol, 256 eq.) and 3 mL of DCM was stirred at room temperature under argon atmosphere for 20 h. Reaction progress was monitored using TLC (CHCl_3_:EtOAc, 1:1 v/v): *R*_f_ = 0.1. Solvent was evaporated under reduced pressure, crude product was dissolved in toluene and evaporated again. Yield: 42.4 mg, 95%; oily residue; ^1^H NMR (400 MHz, DMSO-d_6_): δ 1.41–1.70 (14H, m, cyclooctane, NHCH_2_C*H*_*2*_), 1.79–1.89 (2H, m, cyclooctane), 2.2 (2H, t, *J* = 7.4 Hz, C*H*_*2*_COO), 2.57 (2H, t, *J* = 7.1 Hz, C*H*_*2*_CON), 2.98 (2H, q, *J* = 6.8 Hz, NHC*H*_*2*_), 3.74 (2H, t, *J* = 7.2 Hz, SO_2_C*H*_*2*_), 3.79 (1H, m, C*H* cyclooctane), 6.59 (1H, s, N*H* cyclooctylamine), 8.1 (1H, t, *J* = 5.3 Hz, N*H*CO), 8.33 (2H, s, N*H*_*2*_SO_2_), 10.3 (COO*H*); ^19^F NMR (376 MHz, DMSO-d_6_): δ − 125.01 (C3**-***F*, s), − 133.95 (C5**-***F*, dd, ^1^*J* = 27.7 Hz, ^2^*J* = 11.8 Hz ), -150.6 (C6**-***F*, d, *J* = 26.8 Hz). HRMS (*m/z*): [M]^+^ calcd. for C_21_H_31_F_3_N_3_O_7_S_2_, 558.1550; found, 558.1559.

***4-(3-((2-(cyclooctylamino)-3,5,6-trifluoro-4-sulfamoylphenyl)sulfonyl)propanamido)-N-(3',6'-dihydroxy-3-oxo-3H-spiro[isobenzofuran-1,9'-xanthen]-5-yl)butanamide (GZ20-40).***

Purified with column chromatography (acetone:CHCl_3_, 1:1 v/v). Yield: 17.1 mg, 11%; mp: 180–181 °C; TLC (acetone:CHCl_3_, 1:1 v/v): *R*_f_ = 0.15; ^1^H NMR (400 MHz, DMSO-d_6_): δ 1.41–1.68 (12H, m, cyclooctane), 1.73 (1H, p, *J* = 7.3 Hz, NHCH_2_C*H*_*2*_), 1.80–1.89 (2H, m, cyclooctane), 2.38 (2H, t, *J* = 7.4 Hz, CH_2_CH_2_C*H*_*2*_CON), 2.60 (2H, t, *J* = 7.2 Hz, C*H*_*2*_CONH), 3.07 (2H, q, *J* = 6.7 Hz, NHC*H*_*2*_), 3.76 (2H, t, *J* = 7.1 Hz, SO_2_C*H*_*2*_), 3.76–3.82 (1H, m, C*H* cyclooctane), 6.54 (2H, dd, ^1^* J* = 8.7 Hz, ^2^* J* = 2.3 Hz, C2’’-*H* and C7’’-*H*), 6.59 (2H, d, *J* = 8.6 Hz, C1’’-*H* and C7’’-*H*), 6.60 (1H, d, *J* = 8.7 Hz, N*H* cyclooctylamine), 6.67 (2H, d, *J* = 2.3 Hz, C4’’-*H* and C5’’-*H*), 7.20 (1H, d, *J* = 8.3 Hz, C7’-*H*), 7.81 (1H, dd, ^1^* J* = 8.4 Hz, ^2^* J* = 2.0 Hz, C6’-*H*), 8.14 (1H, t, *J* = 5.6 Hz, CH_2_N*H*CO), 8.33 (1H, d, *J* = 2.3 Hz, C4’-*H*), 8.37 (2H, s, N*H*_*2*_SO_2_), 10.12 (2H, s, O*H*), 10.37 (1H, s, CN*H*CO); ^13^C NMR (100 MHz, DMSO-d_6_): δ 23.28 (cyclooctane), 25.42 (NHCH_2_*C*H_2_), 25.46 (cyclooctane), 27.19 (cyclooctane), 28.33 (*C*H_2_CONH), 32.57 (*C*H_2_COO), 32.71 (cyclooctane), 38.75 (CONH*C*H_2_), 53.17 (SO_2_*C*H_2_), 55.89 (*C*H cyclooctane, d, *J*(^19^F–^13^C) = 11.2 Hz), 83.48 (quaternary *C*), 102.67 (xanthene), 110.20 (xanthene), 113.01 (xanthene), 113.79 (isobenzofuranone), 114.86 (*C1*, dd, ^1^* J*(^19^F–^13^C) = 13.1 Hz, ^2^* J*(^19^F–^13^C) = 5.3 Hz), 124.85 (isobenzofuranone), 126.71 (isobenzofuranone), 127.39 (isobenzofuranone), 128.08 (*C4*, dm, ^1^* J*(^19^F–^13^C) = 16.0 Hz), 129.52 (xanthene), 135.31 (*C2*, d, *J*(^19^F-^13^C) = 13.2 Hz), 137.31 (*C5*, d), 141.26 (isobenzofuranone), 144.41 (*C3*, d, *J*(^19^F–^13^C) = 253.64 Hz), 146.16 (*C6*, dm, *J*(^19^F–^13^C) = 251.9 Hz), 147.08 (isobenzofuranone), 152.36 (xanthene), 159.91 (*C*–OH), 168.31 (*C*OO), 169.11 (*C*ON), 171.96 (CH_2_CH_2_CH_2_*C*ON); ^19^F NMR (376 MHz, DMSO-d_6_): δ -124.96 (C3**-***F*, s), -133.98 (C5**-***F*, dd, ^1^* J* = 27.2 Hz, ^2^* J* = 12.4 Hz ), -150.55 (C6**-***F*, dd, ^1^* J* = 27.0 Hz, ^2^* J* = 6.7 Hz). HRMS (*m/z*): [M]^+^ calcd. for C_41_H_42_F_3_N_4_O_11_S_2_, 887.2238; found, 887.2244.

***3-(cyclooctylamino)-2,5,6-trifluoro-4-((2-hydroxyethyl)sulfonyl)benzenesulfonamide (VD11-4–2*)**^14^.

***2,3,5,6-tetrafluoro-4-((2-hydroxyethyl)sulfonyl)benzenesulfonamide (VD10-35)***^13^.

***2,3,5,6-tetrafluoro-4-((2-hydroxyethyl)thio)benzenesulfonamide (VD10-13*)**^13^.

***4-chloro-N-(2-hydroxyethyl)-2-(phenylthio)-5-sulfamoylbenzamide (EA3-2)***^37^.

***4-(3-(4-fluorophenyl)ureido)benzenesulfonamide (SLC-0111*).** The SLC-0111 compound has been synthesized as previously described^30^**.**

***N-(5-sulfamoyl-1,3,4-thiadiazol-2-yl)acetamide (AZM).*** Acetazolamide has been purchased from Sigma Aldrich, Schnelldorf, Germany.

### Preparation of recombinant CA isozymes

All twelve catalytically active human CA isozymes were prepared recombinantly as previously described. Isozymes CAIX, CAXII, and CAXIV were the catalytic domains of the proteins^14,38^. All isozymes were expressed in *E. coli* except CAIX that has been prepared in mammalian cells. The proteins were chromatographically purified on ion-exchange or benzene sulfonamide containing affinity columns. Protein MW was confirmed by mass spectrometry with 1 Da precision as predicted from gene sequence.

### Determination of compound affinities for recombinant CA isozymes by TSA

Compound affinities for the twelve catalytically active CA isozymes were determined by the thermal shift assay as previously described^30,39^. Protein solutions containing 1,8-anilinonaphthalenesulfonate fluorescent probe were heated from 25 to 100 °C at a rate of one degree per minute while following the fluorescence of the probe. The temperature scanning curves provided the melting temperature *T*_m_ of the protein. The *T*_m_ has a distinct dependence on the concentration of a compound. Stronger binders shift the *T*_m_ to a greater extent than the weak binders. The *T*_m_ dependencies on compound concentration were fit to obtain the affinity *K*_d_^40^.

### Determination of inhibition of CA enzymatic activity by SFA

Inhibition of the CAIX catalytic activity (hydration reaction of CO_2_ to acid protons and bicarbonate) by selected compounds has been determined as previously described^41,42^. The CA enzymatic activity and its inhibition were followed by the rate of change in pH spectrophotometrically followed by the change in color of indicator phenol red. Addition of increased concentrations of an inhibitor slowed the rate of the change in pH. The assay was performed using the stopped-flow equipment. The rate of spontaneous acidification due to CO_2_ hydration in water was subtracted from the enzymatically catalyzed rate.

### Determination of compound binding to CA isozymes by ITC

Affinities of several selected compounds to several human CA isozymes have been determined by ITC as described previously^43,44^. The protein solution was placed in the cell of iTC200 titration calorimeter while the compound solution in identical buffer was placed in the syringe. After thermal equilibration, the compound solution was injected into the cell by identical injections. The heat produced during the reaction was measured by the calorimeter providing the shape of the curve that in turn yielded the enthalpy and the affinity *K*_d_ of the interaction.

### Super-Resolution Radial Fluctuations (SRRF) microscopy in TIRF mode illumination

HeLa cells (ATCC) were cultured in DMEM medium (Thermo Fisher) supplemented with 10% FBS, 2 mM glutamine, 100 U/mL penicillin, and 100 µg/mL streptomycin at 37 °C in humidified atmosphere, containing 5% CO_2_. Three days before imaging experiments, cells were seeded at the density of 25,000 cells per well onto an 8-well CG imaging chambers (Zell Kontakt GmbH; Nörten-Hardenberg, Germany) and cultivated in conditions of normoxia (21% O_2_ and 5% CO_2_ in cell incubator) or hypoxia (1% O_2_ and 5% CO_2_ supplied by gas controller into incubator of Cytation 5 Imaging system (BioTek; Winooski, VT, USA)). To visualize CAIX, live cells were incubated with the H7 antibody (dilution 1:100 in FluoroBright DMEM (FB) (Thermo Fisher)) in CO_2_ incubator for 30 min. After washing 3 × 5 min with PBS at room temperature (gently rocking), the cells were incubated w/ secondary Texas-Red-conjugated anti-mouse antibodies (dilution 1:100 in FB) in CO_2_ incubator for 30 min. After washing 3 × 5 min with PBS at room temperature, the cells were incubated with GZ19-32 or GZ19-37 (2 or 10 nM in FB) for 5 min and washed 3 × 3 min with PBS. The PBS was replaced by FB supplemented with 10 µM nuclear stain Hoerchst 33,342 (Thermo Fisher). After 10 min, the cells were imaged by fluorescence microscopy.

Imaging was carried out with the microscopy setup as described in^45,46^. Briefly, live cell widefield epifluorescence and TIRF were conducted using an inverted microscope built around a Till iMIC body (Till Photonics/FEI; Munich, Germany), equipped with UPLSAPO 20 × oil (NA 0.85) and TIRF APON 60 × oil (NA 1.49) objective lenses (Olympus Corp., Tokyo, Japan). The samples were sequentially excited with 405 nm (150 mW), 488 nm (100 mW) PhoxX laser diodes (Omicron-Laserage; Rodgau, Germany) or AOTF controlled 561 nM (106 mW) Cobolt Jive DPSS laser (Cobolt AB, solna, Sweden) combined in the SOLE-6 light engine (Omicron-Laserage). Excitation light was launched into Yanus scan head, which along with a Polytrope galvanometric mirror (Till Photonics/FEI; Munich, Germany) was used to position the laser for widefield epifluorescence or azimuthal 360° TIRF (approx. 100 nm depth) illumination. Excitation and emission light was spectrally separated with the imaging filter cubes containing a 390/40 BrightLine exciter filter (Semrock, NY, USA), a zt 405 rdc 2 mm beamsplitter (Chroma Technology Corp, Vermont, USA), and a ET 460/50 emitter filter (Chroma) for Hoechst 33,342 staining or containing a zt 488/561 rpc 2 mm dual-line beamsplitter (Chroma) and a 524/628 BrightLine dual-band bandpass emission filter (Semrock; West Henrietta, NY, USA) for GZ compounds (excited with 488 nm laser) or Texas-Red-conjugated antibodies (excited with 561 nm laser). Additionally, the brightfield channel was used for the determination of cell borders. The electron-multiplying charge-coupled device Ultra 897 camera (Andor Technology; Belfast, UK) was mounted to a microscope through a TuCam adapter with 2 × magnification (Andor Technology). The camera was cooled down to − 100 °C with the assistance of a liquid recirculating chiller Oasis 160 (Solid State Cooling Systems; Wappingers Falls, NY, USA). All measurements were performed in the 8-well CG imaging chambers, and at least 5 selected areas per well were captured as 16-bit OME-TIFF multicolor Z-stacks (100 frames, with 200 nm piezo-focusing increment), with 100 ms exposure time and EM gain 300 in the case of widefield epifluorescence imaging or two-color time-stacks (1000 frames at 15 Hz) in the case of TIRF/SRRF experiments. For different fluorescent ligands and stains, the laser powers were varied to enable optimal signal levels (the absence of detectable crosstalk signal in the channels was ensured). The widefield epifluorescence Z-stacks were deconvolved with EpiDEMIC plugin^47^, the objects were segmented with Spot Detection plugin in an ICY platform^48^, and the degree of multichannel co-localizations were estimated by Mander’s coefficients using Colocalization Studio^49^. The SRRF images were reconstructed with GPU of NVIDIA Quadro P4000 video card, using Fiji ImageJ 1.53i with NanoJ-SRRF plugin 1.14 ^50^ (1000 frames per time point; temporal analysis settings: Temporal Radiality Auto-Correlations; radiality magnification: 5; symmetry axes: 8, ring radius 0.5). For each fluorescent ligand at each concentration, at least two independent experiments were performed.

### Cell culture for immunofluorescent staining and microscopy

Human cervical adenocarcinoma cells (HeLa) were kindly provided by Dr. A. Kanopka (Vilnius University) and lung carcinoma cells (A549) were kindly provided by Dr. V. Petrikaitė (Vilnius University). Cells were cultured in Dulbecco’s Modified Eagle’s Medium (DMEM) with GlutaMAX™ (Gibco, ThermoFisher) supplemented with 10% fetal bovine serum (ThermoFisher) in a humidified atmosphere at 37 ℃ and 5% CO_2_. Cells were exposed to hypoxic conditions in the humidified CO_2_ incubator with oxygen control (Binder, Germany), and conditions were 1% O_2_, 5% CO_2_ and 94% N_2_ and 37 ℃. Both cell lines were regulary checked for mycoplasma contamination using SouthernBiotech™ Mycoplasma Detection Kit (# OB1310001) according to manufacturer’s directions.

### Immunofluorescent staining and microscopy

**Staining of fixed cells**. HeLa WT and HeLa-CAIX-KO cells were cultured on the glass coverslips in 12-well plates under hypoxia for three days. Cells were fixed by submerging glass coverslips into 20 ℃ methanol for at least 5 min. To visualize CAIX, live cells were rehydrated and stained using H7 antibody^31^ diluted 1:200 in PBS, supplemented with 0.05% Tween-20 (PBS-Tw) in 37 ℃ incubator for 30 min. After washing 3 × 10 min with PBS-Tw at room temperature (RT), cells were incubated with secondary Alexa Fluor 488-conjugated anti-mouse antibodies (Cat # A-11034, ThermoFisher), dilution 1:200 in PBS-Tw, in 37 ℃ incubator for 30 min. After washing 3 × 10 min with PBS-Tw at RT, glass coverslips were briefly submerged into distilled water and mounted on a glass slide cells down on a drop of ProLong™ Gold Antifade Mountant with DAPI. Cells were observed using an automated fluorescence microscope EVOS FL Auto (ThermoFisher).

**Staining of live cells**. HeLa cells were cultured in 12-well plates under normoxia or hypoxia for three days. To visualize CAIX, live cells were incubated with purified H7 antibody^31^ in the CO_2_ incubator for 30 min. The 1.13 mg/ml antibody stock solution was used at a dilution 1:100 in FluoroBright (FB)(ThermoFisher) medium. After washing 3 × 5 min with PBS at RT (gently rocking), cells were incubated with secondary Alexa Fluor 594-conjugated anti-mouse antibodies (Cat # A-21203, ThermoFisher), dilution 1:100 in FB, in CO_2_ incubator for 30 min. After washing 3 × 5 min with PBS at RT, cells were incubated with GZ19-32 (10 nM in FB) for 5 min at room temperature and washed 3 × 3 min with PBS. PBS was replaced by FB and a drop of NucBlue Live ready probe (ThermoFisher) was added into the wells. After 10 min cells were observed using an automated fluorescence microscope EVOS FL Auto (Thermo Fisher).

### Making of CRISPR-Cas9 knockout of CAIX in HeLa cells

GeneArt CRISPR Nuclease vector (Invitrogen) was used for the disruption of CAIX gene in Hela cells. Three pairs of gRNA oligomers: fwd. GATGCAGGAGGATTCCCCCTGTTTT, rev. AGGGGGAATCCTCCTGCATCCGGTG; fwd. ACCCCAGAATAATGCCCACAGTTTT, rev. TGTGGGCATTATTCTGGGGTCGGTG; fwd. TGAAGTGACTCCACAAGGGTGTTTT, rev. ACCCTTGTGGAGTCACTTCACGGTG, were annealed to make 3 double-stranded oligos which were subcloned into linearized GeneArt CRISPR Nuclease vector. The obtained contructs were used for co-transfection of HeLa cells using TurboFect transfection reagent (ThermoFisher) according to the manufacturer’s protocol. Transfection was performed for only 6 h to avoid cytotoxicity. Transfected cells were cultured in fresh medium for 48 h and then transferred to 96 well plates for single cell colony formation. No cell sorting was performed because thransfection efficiency of HeLa cells by TurboFect reaches 70–90%. After 3 weeks, colonies were checked for CAIX gene disruption. Genomic PCR was performed using Phire Tissue Direct PCR Master Mix (ThermoFisher) according to the manufacturer’s protocol. Sequencing of PCR products confirmed the presence of deletions in CAIX gene. A clone chosen for further work contained a large deletion (413–6941 nt.) in one of the alleles and 2 small deletions (163–192 nt. and 412–415 nt.) in the other allele. A 4 nt. deletion (412–415) caused a frame shift, leading to a meaningless gene translation and thus gene inactivation. The absence of CAIX protein in HeLa-KO cells was confirmed by Western blotting, flow cytometry, and immunofluorescence staining using three types of antibodies that recognize human CAIX.

#### Western blotting

Hela WT and HeLa-CAIX-KO cells were cultured in hypoxia for 72 h, harvested by scraping, centrifuged and extracted in RIPA buffer with 1 mM PMSF on ice for 30 min. After high speed centrifugation, the supernatants were mixed with SDS sample buffer, separated by SDS–polyacrylamide gel electrophoresis and transferred to nitrocellulose membranes (Carl Roth). Membranes were stained with Ponceau S solution (0.1% w/v in 5% acetic acid) and cut into the strips, corresponding to each loading well. All incubations with antibodies were performed in 5 ml tubes, while washing was done in 10 cm Petri dishes. Nonspecific binding was blocked by 3% bovine serum albumin (BSA) in PBS, containing 0.05% Tween-20 (PBS-Tw) for 30 min at RT. Monoclonal M75 antibody (BioScience, Slovakia) was used at the dilution 1:200 and β-tubulin Monoclonal Antibody (ThermoFisher, #32–2600) was used at the dilution 1:2000. Antibodies were diluted in PBS-Tw supplemented with 1% BSA and 0.02% sodium azide and membranes were incubated with slow shaking at RT overnight. After washing in PBS-Tw, goat anti-mouse IgG antibody conjugated with alkaline phoshpatase (Merck Millipore, # 69,266) were used at the dilution 1:1000 in PBS-Tw with 1% BSA for 1 h at RT. After washing with PBS-Tw and PBS (final wash), protein bands were visualized using 1-Step NBT/BCIP Substrate Solution (Thermo Fisher). Color development reaction was stopped by transferring membranes to cold distilled water.

#### Flow cytometry

Hela WT and CAIX-KO cells were cultured in 6-well plates in DMEM uder 21% O_2_ versus 1% O_2_ conditions for 48 h. Cells were harvested, washed, resuspended in FACS buffer and stained for 30 min using anti-human CAIX AF488 antibody (R&D Systems, Cat FAB2188G) and Mouse IgG2A Alexa Fluor 488-conjugated Antibody (R&D Systems, cat. IC003G) as an isotype control (ISO). At the end, 7AAD staining was added for live cell separation. After centrifugation, the cells were suspended in 200 μl of FACS buffer and live cells were analyzed by flow cytometry using BD FACS Canto II system. Data were analyzed using FlowJo. The mean fluorescence intensity (MFI) values are shown as averages of two experiments.

#### Determination of CA activity by gas-analysis mass spectrometry

Catalytic activity of CA in hypoxic human cervical adenocarcinoma (HeLa) cells was determined by monitoring the ^18^O depletion of doubly labeled ^13^C^18^O_2_ through several hydration and dehydration steps with a quadrupole mass spectrometer (OmniStar GSD 320; Pfeiffer Vacuum, Asslar, Germany) as previously described^16,51^. HeLa cells were cultured in Dulbecco’s Modified Eagle’s Medium–high glucose (D5671; Sigma Aldrich, Schnelldorf, Germany), supplemented with 10% fetal calf serum and 1% penicillin / streptomycin. The cell line was cultured under hypoxia (1% O_2_) for three days prior to measurements. Cells were detached from the plate with Accumax Cell Detachment Solution (ACM-1F; Capricorn Scientific, Ebsdorfergrund, Germany), washed and resuspended in HEPES-buffered saline (143 mM NaCl, 5 mM KCl, 2 mM CaCl_2_, 1 mM MgSO_4_, 1 mM Na_2_HPO_4_, 10 mM HEPES, pH 7.2). Approximately 3 × 10^6^ cells, dissolved in 5 mL of HEPES-buffered saline (pH 7.2, RT), were used for every single measurement. To ensure an equal amount of cells within one set of measurements cells were pooled and then aliquoted according to the number of tested inhibitor concentrations. The cell suspensions were pre-incubated with the inhibitor for up to three hours. For every measurement, the non-catalyzed reaction was determined for 12 min in the presence of inhibitor, before cell suspension was added to the measuring cuvette and the catalyzed reaction was determined for 12 min. For every batch of cells, CA activity in the presence of inhibitor was normalized to the activity in the absence of inhibitor. *IC*_*50*_ values were determined by Hill1 fit using OriginPro 8.6 (OriginLab Corporation). CA enzymatic activity in units (U) was calculated as defined by Badger and Price^52^. From this definition, one unit corresponds to 100% stimulation of the non-catalyzed ^18^O depletion of doubly labeled ^13^C^18^O_2_.

#### Cell viability assay for CA activity by gas-analysis mass spectrometry

Potential cytotoxic effects on HeLa cells were determined by XTT assay. The XTT was carried out as previously described by Scudiero et al.^53^. HeLa cells were cultured under hypoxia (1% O_2_) for three days prior to measurements. Cells were detached from the plate with Accumax Cell Detachment Solution (ACM-1F; Capricorn Scientific, Ebsdorfergrund, Germany), washed and resuspended in HEPES-buffered saline (143 mM NaCl, 5 mM KCl, 2 mM CaCl_2_, 1 mM MgSO_4_, 1 mM Na_2_HPO_4_, 10 mM HEPES, pH 7.2). For each measurement, 100,000 cells were incubated with 1 nM or 1 µM of GZ18-23 for 3 h prior to adding the XTT (No. 38450, SERVA Electrophoresis GmbH, Heidelberg, Germany) / electron coupling reagent (PMS; No. 32030, SERVA). XTT-specific absorbance was measured at 492 nm using a microplate reader (Tecan Infinite F200; Tecan Trading AG, Männedorf, Switzerland). Background absorption was measured at 690 nm. Cells, which were not incubated in GZ18-23, were used as control (100%).

#### Fluorescein-labeled compound dosing experiments

HeLa cell culture was grown in 12-well plates, either under hypoxia (1% O_2_) or under normoxia (21% O_2_), for 70 to 76 h. The medium was removed. Then 200 µL of serially diluted compound (twelve twofold steps) in the FB medium, usually starting at 80 nM (80, 40, 20, …, 0 nM), was added and incubated at 37 ℃ under normoxia in the CO_2_ incubator for 20 min (2 or 5 min in some experiments where specified). Then the compound solution was removed and the cells were washed 3 times for 1.5–2 min with 400 µL of PBS at RT. Then 180 µL of TrypLe express enzyme (ThermoFisher) was added to each well and incubated in CO_2_ incubator for 5 min to 10 min until the cells deattached from the surface. After addition of 20 µL of Defined Trypsin Inhibitor solution (ThermoFisher), cells were resuspended by pipetting and 150 µL of the suspension from each well were transferred to black Thermo Scientific™ Nunc MicroWell 96-Well Optical-Bottom Plates for fluorescence and absorbance measurements. The fluorescence was recorded at 485 nm excitation and 520 nM emission wavelengths in the plate reader (Synergy HTX, BioTek). The absorbance was recorded at 650 nm to measure the optical density of the cell suspension.

The compound solution was prepared by weighing 3–5 mg of a purified compound and dissolving in DMSO at 10 mM concentration. The 1 mM compound stock in DMSO was prepared by taking 10 µL of 10 mM stock and adding to 90 µL DMSO. The 10 µM compound stock was prepared by taking 10 µL of 1 mM compound stock in DMSO and adding 990 µL of the FB medium (this stock contained 1% of DMSO). Then 80 nM compound solution was prepared by taking 10 µL of 10 µM solution and adding 1.24 mL of the FB medium and placed to the first Eppendorf tube. Then 300 µL of FB medium were added to the Eppendorf tubes 2 to 12. A serial dilution was performed by taking 300 µL of solution from tube 1 to 2, then from 2 to 3 and so on while mixing vigorously at each step. No solution was transferred to the last tube thus it contained only FB medium and no compound. Then 200 µL from 12 tubes were transferred to the 12 wells of cell culture-containing 12-well plate, starting from the lowest concentration. Several different dilution series were prepared in various experiments beginning with 40 nM, 80 nM, 160 nM, 80 µM, or 160 µM depending on compound affinity using the scheme described above.

#### Unlabeled compound competition experiment with the fluorescein-labeled compound

The same compound solutions and cells were used in the competition experiments as in the dosing experiments. The 20 nM solution of GZ19-32 was prepared by adding 6 µL of 10 µM solution of GZ19-32 in 1% DMSO and 3 mL of the FB medium. A serial twofold, twelve step dilution is prepared of the unlabeled compound. Same volumes of 20 nM GZ19-32 were added to the twelve serially diluted compound solutions and mixed. The obtained mixture is then applied to the cells grown in 12-well culture plates, starting from the lowest concentration of unlabeled compound. Incubation, washing and fluorescence measurements were performed the same way as in the compound dosing experiments. In the dosing experiments, the concentration of compound was as listed. However, in the competition experiments, the compound concentrations were lower by a factor of two because equal volumes of 20 nM GZ19-32 (in most experiments) and competing compound solutions were prepared and applied to cells as a mixture. The concentration of DMSO was constant in the serially diluted solutions.

Usually, the experiment was performed for 1.5 h at RT (20 ℃) under normoxic conditions. However, where specified, the experiment has been performed entirely under hypoxic conditions in Ruskin InvivO_2 _200 workstation to make sure that the experiment performed in normoxia and hypoxia yielded the same binding constants (Figure S6).

#### Application of the single-site binding model for the compound dosing curves

Application of fluorescently labeled ligand GZ19-32 to cancer cells expressing CAIX will stain the cells in dose-dependent manner. The fraction of saturation increases from 0 to 1. The fraction of bound GZ19-32 (*f*) is equal to the molar concentration of bound GZ19-32 per molar concentration of total available CAIX, the so-called total protein $P_{t}$.

$$ f = \frac{{\left[ {{\text{GZ}}19 - 32} \right]_{{{\text{bound}}}} }}{{P_{t} }} $$

Upon GZ19-32 dosing, the observed fluorescence signal *F* can be modeled to change from the minimal background signal ($F_{min}$) to maximal saturated value ($F_{max}$):

$$ F = F_{\min } + \left( {F_{\max } - F_{\min } } \right)f $$

Application of a single-site binding model (Morrison equation) yields the bound fraction as:

$$ f = \frac{{K_{d} + P_{t} + L_{t} - \sqrt {\left( {K_{d} + P_{t} + L_{t} } \right)^{2} - 4P_{t} L_{t} } }}{{2P_{t} }} $$

where the $K_{d}$ is the equilibrium dissociation constant of the fluorescently-labeled ligand and *L*_t_ is the total added ligand concentration.

This equation was fit to the dosing curves of several fluorescently-labeled ligands to both the cells grown in hypoxia and in normoxia, both to the WT and CRISPR-Cas9 CAIX knockout cells. The cells bound both the GZ19-32 and GZ20-40 ligands that differed only in the length of the linker and both contained the same head-group. However, the GZ19-31 ligand, that contained the CAI-selective head-group, did not bind to the cells. Furthermore, the ligands that had their sulfonamide amino group substituted with the methyl completely abandoned their capability of binding CAIX in this concentration region. The binding was also abolished for the CAIX-KO HeLa cells. However, at larger, micromolar, concentrations, all ligands bound to all cells indicating that a low-affinity binding occurred in this concentration region.

#### Application of the competition model for the determination of various compound ***K***_d_ values for cell-expressed CAIX

The model of ligand binding to a receptor in the presence of another competing ligand was derived in 1995^54^ and also has been applied to ITC^55^. According to it, the fraction of fluorescently labeled GZ19-32 (ligand B) bound to CAIX in the presence of any competing ligand A can be calculated using the equation:

$$ f_{{{\text{GZ}}19 - 32}} = \frac{1}{{P_{t} }}\frac{{L_{{t.{\text{B}}}} \left( {2\sqrt {a^{2} - 3b} \cos \left( {\frac{1}{3}\arccos \frac{{ - 2{\text{a}}^{3} + 9{\text{ab}} - 27{\text{c}}}}{{2\sqrt {\left( {{\text{a}}^{2} - 3{\text{b}}} \right)^{3} } }}} \right) - a} \right)}}{{3K_{{d,{\text{B}}}} + \left( {2\sqrt {a^{2} - 3b} \cos \left( {\frac{1}{3}\arccos \frac{{ - 2{\text{a}}^{3} + 9{\text{ab}} - 27{\text{c}}}}{{2\sqrt {\left( {{\text{a}}^{2} - 3{\text{b}}} \right)^{3} } }}} \right) - a} \right)}} $$

where

$$ a = K_{d,A} + K_{d,B} + L_{t,A} + L_{t,B} - P_{t} $$

$$ b = K_{d,A} \left( {L_{t,B} - P_{t} } \right) + K_{d,B} \left( {L_{t,A} - P_{t} } \right) + K_{d,A} K_{d,B} $$

$$ c = - K_{d,A} K_{d,B} P_{t} $$

where $L_{t,A}$ and $L_{t,B}$ are the molar concentrations of added ligands A and B, respectively. The $K_{d,A}$ and $K_{d,B}$ are the equilibrium dissociation constants of ligands A and B.

We made a competition (displacement) experiment, where the concentration of the GZ19-32 fluorescently labeled ligand was kept constant at 10 nM and the concentration of the unlabeled ligand (VD11-4–2 or any other ligand whose affinity for CAIX may not be known) was varied from 0 to 80 nM (by performing serial 2 × dilutions from 80 nM downwards with the last sample concentration 0 nM as explained above), we obtained a competitive dosing curve, where the unlabeled ligand out-competed the fluorescently-labeled one at sufficiently high concentrations.

This result was obtained only if we applied the mixture of both ligands onto the cells. However, if the cells were pre-saturated with GZ19-32 fluorescently labeled ligand for 20 min and then the competing unlabeled VD11-4-2 was applied, there was essentially no decrease in fluorescence occurring, and the competition did not occur. The unlabeled ligand was unable to displace the labeled one. This is explained by the long residence time of both ligands. We have previously shown by SPR that VD11-4-2 had 5.5 h t_1/2_ residence time on CAIX^33,34^. Therefore, the equilibrium could not be achieved in 20 min when both ligands were present. However, if both ligands are applied as a mixture, an approximate equilibrium is achieved based on diffusion rates of labeled and unlabeled ligands.

## Discussion

The role of CAIX in cancer is still not fully understood, despite numerous studies. As shown here, the CRISPR-Cas9 knockout of CAIX in HeLa cells led to a cell line that despite a slower growth under hypoxic conditions than the WT was still able to grow in the absence of CAIX. This result may indicate that even full inhibition of CAIX in human cancers may not completely stop cancer progression due to alternative growth pathways by potentially over-expressing other membrane CA isozymes such as CAXII or CAXIV. The CAIX-KO cell line should be further studied to understand the role of CAIX better.

The inhibition of CAIX may be still beneficial in treating CAIX-expressing cancers because it would reduce the acidification in the tumor microenvironment that has been generated by the high amount of CAIX present under hypoxic conditions. The easiest way to reduce the acidification is to apply chemical compounds–inhibitors of CAIX. There are two difficulties in the design of the inhibitors. First, the inhibitors must possess a sufficiently high affinity for CAIX. Second, the inhibitors must possess a sufficiently low affinity for the remaining 11 isozymes of CA or any other proteins in the human body. In other words, there should be sufficient selectivity and specificity in binding CAIX over other CAs.

In this work, we analyzed the binding of inhibitor GZ18-23 that has been demonstrated to reach pM affinity for recombinant CAIX and significant selectivity over the remaining 11 catalytically active human CA isozymes. The compound had significantly improved aqueous solubility and pharmacokinetic properties as compared to earlier lead VD11-4-2^14^. To visualize the location of this compound in cancer cells, attachment of a fluorescent group to a lead compound that would both visualize and quantify the binding.

An important factor in the design of CAIX inhibitors is the concentration of CAIX molecules in the tumor. Our results here show that the concentration of CAIX in the cell culture was between 1 and 20 nM depending on the growth stage. This means that no matter how strongly the drug binds the CAIX there would be a need to have 20 nM concentration of the inhibitor to saturate and fully inhibit the enzyme, whose concentration is 20 nM. At such a high concentration, the inhibitor does not necessarily need to possess picomolar affinity. However, greater affinity and slower off-rate would be highly beneficial in achieving as complete inhibition of CAIX as possible.

The inhibitor GZ18-23 possessed 30 pM *K*_d_ for CAIX and 1 nM *K*_d_ for CAXII. Thus there was 30-fold selectivity for CAIX over CAXII. Such inhibition of CAXII may be beneficial since CAXII has also been implicated in cancer progression^56–58^. Thus the compound may serve as a cancer related-CA specific inhibitor. Furthermore, GZ18-23 possessed 100-fold selectivity over CAXIV, 300 × over CAXIII, 1000 × over CAVII, and more than 3000 × over the remaining CA isozymes. Notably, the selectivity over the ubiquitous isozyme CAI reached 150-thousand-fold.

For comparison, acetazolamide possesses only 2 × selectivity over CAII, 3 × selectivity over CAVII and CAXIV, and 100 × selectivity over the ubiquitous CAI. Such selectivities are relatively low and lead to undesired inhibition of other CA isozymes in addition to CAIX and thus elevated toxicity. Furthermore, the relatively low affinity requires a high dose of the inhibitor to saturate the CAIX while also nearly saturating numerous other isozymes.

Application of fluorescein-labeled compounds has previously been performed while administering rather high, micromolar concentrations of compounds^10,11,23^. Our results here indicate non-specific binding of various compounds to unknown targets other than CAIX when applying the fluorescein-labeled inhibitors at concentrations higher than approximately 200 nM to 2 µM. The fluorescent compounds bound to all cells, including the cells that have been grown under normoxic conditions and the cells that had CAIX knocked-out. Thus the compounds when applied at higher concentrations than 200 nM bound to targets other than CAIX. Still, these observations may not apply to previously used fluorescein-labeled compounds due to different chemical structures.

At high concentrations, the fluorescein-labeled compounds likely bound not only to the other CA isozymes but also to other proteins because the compounds that had the sulfonamide amino group substituted with the methyl and thus did not possess the capability of recognizing the CA via the sulfonamide-Zn(II) coordination bond still bound to the cells grown both under hypoxic and normoxic conditions and also to the CAIX-KO cells (Fig. 9). Thus, at concentrations exceeding 1 µM, all fluorescein-labeled compounds used in this study also most likely bound to the targets other than CAIX. Therefore, we suggest that the fluorescent compound application should be performed in the 1 to 10 nM concentration range to avoid all the above-mentioned non-specific effects.

One of the importances of this study is the determination of common CA inhibitor affinities for cellular CAIX. The technique that involves the competition of the studied compound with a high affinity for CAIX fluorescein-labeled compound GZ19-32 showed that the *K*_d_ for a series of compounds matched the affinities determined by the thermal shift assay (TSA) and other assays for the recombinantly prepared catalytic domain of CAIX. The competitive binding model has been applied for the first time for CAIX inhibitors in live cells to the best of our knowledge.

Another important finding of this study is that the dosing curves as shown in Fig. 8 do not directly indicate the affinity of the inhibitor. The midpoint of the sigmoid of the curve position relative to the abscissa axis may indicate the *K*_d_ of the fluorescein-labeled compound. It may seem that the curves shifted to the left indicate stronger binding relative to the unshifted ones. However, our results showed that the midpoints (around 10 nM) did not indicate the *K*_d_. The actual *K*_d_ was around 100 pM. The position of the curve instead indicated the concentration of the CAIX enzyme in the system. A shift to the right in the position showed an increase or decrease of the produced CAIX concentration. In addition, the shift to the right was also associated with an increased plateau of the curve on the ordinate axis. The saturation occurred at higher concentrations of the fluorescein-labeled inhibitor as illustrated in Fig. 10.

The precision of the dissociation constant is often overestimated while the error is usually underestimated. Quite often, scientists claim that the error is around 5 or 10% of the *K*_d_ value. However, it is important to remember that the *K*_d_ is logarithmically related to the change in Gibbs energy upon binding. The Gibbs energy distributes almost normally while the *K*_d_ has a log-normal distribution and thus the error of *K*_d_ should not be expressed as ± value, but instead it should be stated that the error of *K*_d_ is ‘two-fold’ or some other value^59^. Thus if the *K*_d_ has been determined to be 10 nM, and if the error is ‘two-fold’ then the error should be expressed in the confidence intervals between 5 and 20 nM (values are twice smaller and twice greater than the measured value). In our experience, various techniques yield a precision of around twofold in best cases. Greater precisions are very hard to achieve and they require multiple repetitions of the experiments. Our measurements yielded the precision of 1.6 to threefold. Therefore, if the values differ by a factor of 2 they should be considered within an error margin.

When comparing the *K*_d_ obtained by TSA of compound binding to recombinantly prepared catalytic domain of CAIX with the *K*_d_A values (Table 2) obtained for compound binding to CAIX expressed on live cell surface, from the competitive binding model, we see that the values nearly perfectly match each other. Furthermore, the same affinities were demonstrated both for CAIX expressed on both HeLa and A549 cell surface. However, the SLC-0111 appeared to bind CAIX 6-times weaker in A549 than HeLa cells. The reason for this difference is unclear, but insignificantly exceeds the error margin. The strongly-binding compounds, VD11-4–2 and GZ18-23, yielded slightly lower affinities for CAIX on cells than for the purified CAIX. The difference reached a factor of 3 to 10. This may indicate that the most strongly binding compounds actually bind systematically weaker to cell-expressed CAIX than to recombinantly prepared CAIX due to the long residence time of the inhibitors of approximately 5 h thus unable to reach the complete equilibrium^33^.

The competition experiment appeared to not depend on the hypoxic conditions for the duration of the experiment after taking the cells out of the hypoxic camera where the cells have been grown for 72 h. The competition experiment lasted for approximately 3 h and the *K*_d_ were the same when the whole experiment has been performed in the hypoxic chamber and when it was performed ‘on the bench’.

Here we have determined the *K*_d_ of compound binding to CAIX by the competitive assay and TSA (also termed FTSA—fluorescent thermal shift assay). However, numerous inhibitors are described in the literature using the stopped-flow assay (SFA) measuring the inhibition of CA enzymatic activity^60^. We prefer TSA over SFA because of the wider dynamic range. The SFA cannot determine compound affinities in the pM range because in the SFA the concentration of CAIX is usually around 10 nM. Therefore, all inhibitors with the IC_50_ below this value will appear to have the IC_50_ of 5 nM, half of the concentration of the enzyme. Thus it is impossible to determine the inhibitor affinities that are stronger than 5 nM^61^. Application of the Cheng-Prusoff equation to obtain the *K*_i_ from *IC*_50_ depending on the *K*_M_ of the isozyme and the CO_2_ concentration may reach a value of 1–2 nM, but the pM range is still not accessible^29^. Therefore, in our opinion, TSA is a preferred assay over SFA to be used in the inhibitor design.

On the other hand, the TSA does not have this limitation and can determine pM and greater affinities in the same assay with weak mM inhibitors. The enzyme concentration here is 10 µM, but this does not limit the determination of the melting temperature. However, we argue that it is important to use as many assays for affinity determination as possible. There are numerous assays available such as TSA, SFA, isothermal titration calorimetry (ITC), surface plasmon resonance (SPR), microscale thermophoresis (MST), and the mass-spectrometry-based assay. We have previously shown that TSA, SFA, and SPR assays yield the same *K*_d_ within the error margin^33,41^ under the condition that both assays are applied in the correct concentration range of approximately 10 nM to 1 mM. However, all assays have advantages and limitations. Here we have introduced an additional assay where the affinity of compounds for CAIX on the cell surface can be determined via competition with the fluorescein-labeled CAIX-specific ligand.

The staining of live HeLa cells with the fluorescein-labeled compound provided a picture of CAIX distribution on the cell surface. We confirmed previous observations that the CAIX is preferably expressed on invadopodia^62,63^. In addition, the SSRF/TIRF microscopy and deconvoluted Z stacks of widefield microscopy showed that the CAIX is expressed on the cell surface in a distinctly separated conglomerates consisting of at least 10 molecules of CAIX. These conglomerates are separated by 0.7 to 1.8 µm. The function of such agglomerate is not clear but the observation was confirmed by near-perfect colocalization of fluorescein-labeled compounds with the H7 antibody. Agglomerates may be related to the focal points because CAIX has been demonstrated to be associated with focal contacts and cell adhesion^64^. It was necessary to use super-resolution microscopy for the demonstration of the co-localization. Based on the estimate of bound GZ19-32 molecules and if the conglomerates are separated on an average 1 µm distances evenly distributed throughout the cell surface, the numbers indicate that there should be approximately 100 CAIX molecules in each agglomerate. One possible explanation could be that the places of high concentration of CAIX on cell membrane are related to protein clustering in lipid rafts^65^.

It should be kept in mind that the *K*_d_ values determined by the competitive assay were the *observed* ones, and they were not *intrinsic*. When sulfonamide ligands bind to any CA isozymes containing the Zn(II), they undergo a binding-linked deprotonation of the sulfonamide group. The CA isozymes also may undergo binding-linked protonation of the hydroxide bound to the Zn(II) in the unliganded form of the enzyme. It is important to dissect these linked reactions in order to study the relationships between the compound structure and the affinity for the enzyme. We consider the concept of the intrinsic affinities to be an important part of the structure-based drug design.The calculation of the intrinsic binding constants has been previously described^33,66^ and this model fully applies to the binding of the fluorescein-labeled compounds described here. These compounds can be widely used for the discovery and design of novel CAIX inhibitors for anticancer therapy.

## Conclusions

A series of designed fluorescein-labeled compounds recognized the CAIX expressed on the surface of live cancer cells with the same affinity as the recombinant CAIX. Control compounds that bear methyl-displaced headgroup or a non-CAIX selective group did not bind cellular CAIX in the 10 nM concentration range and could be clearly distinguished from CAIX-selective probes. The CAIX-CRISPR-Cas9 knockout cells and the cells grown under normoxia did not express CAIX and thus did not bind the CAIX-selective probe. However, non-specific binding was observed above 1 µM concentration. The probes could be used for time-dependent quantification of CAIX expression, visualization of CAIX on the cell surface and also for the determination of commonly used CAIX inhibitor affinities for cellular CAIX via the competition model of the fluorescein-labeled compounds.

## Supplementary Information

Supplementary Information.

## Data Availability

All data generated or analysed during this study are included in this published article and its supplementary information files.

## Abbreviations

A549: Human lung adenocarcinoma cells
CA: Carbonic anhydrase
CAIX: Carbonic anhydrase isozyme IX
CAIX-KO: CAIX knockout cells
FB: FluoroBright medium
FTSA: Fluorescent thermal shift assay (also termed DSF—differential scanning fluorimetry)
HeLa: Human cervical adenocarcinoma cells
ITC: Isothermal titration calorimetry
SFA: Stopped-flow assay of determining inhibition of CA enzymatic activity
SPR: Surface plasmon resonance
SRRF: Super-resolution radial fluctuations
TIRF: Total internal reflection fluorescence
TSA: Thermal shift assay
WB: Western blot
WT: Wild type
