# Supplementary material for: Picomolar fluorescent probes for compound affinity determination to carbonic anhydrase IX expressed in live cancer cells
Source: Sci Rep. 2022 Oct 21;12:17644. doi: 10.1038/s41598-022-22436-1 (PMC9586938; doi:10.1038/s41598-022-22436-1)
Supplement: Supplementary file 1 — Supplementary Information. [file 41598_2022_22436_MOESM1_ESM.pdf]

Supplementary Materials

Picomolar Fluorescent Probes for Compound Affinity Determination to Carbonic Anhydrase IX Expressed in Live Cancer Cells

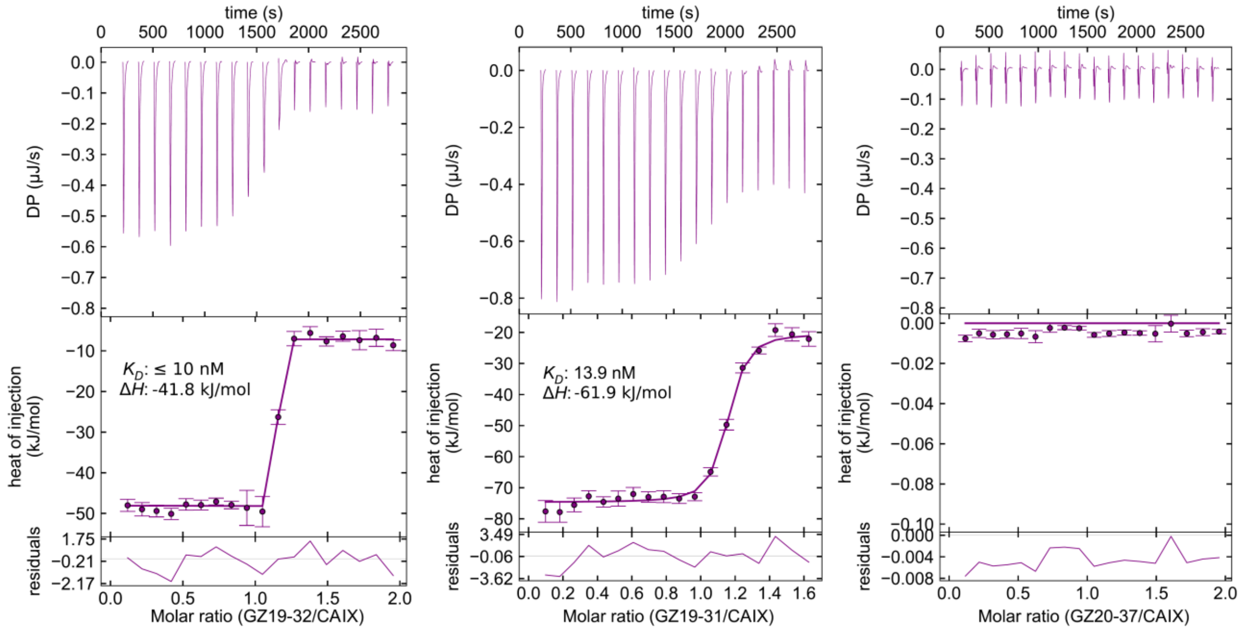

Figure S1. Isothermal titration calorimetry (ITC) measurements of recombinant human CAIX interaction with compounds GZ19-32 (left), GZ19-31 (middle), and GZ20-37 (right). The binding affinity of GZ19-32 was too high to be accurately measured by ITC due to the limitation related to the Wiseman parameter *C*.

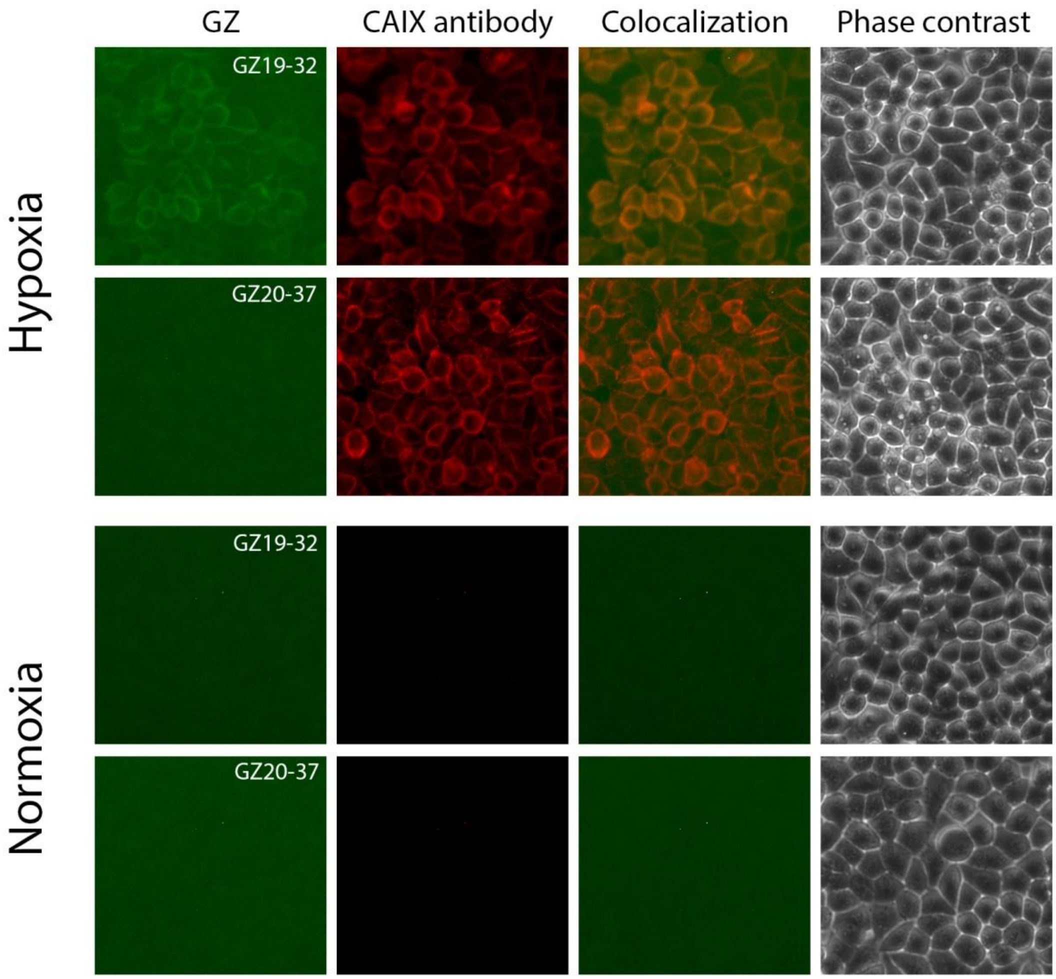

Figure S2. Images of the HeLa cells (WT) incubated for 5 min with 10 nM fluorescein-labeled compounds GZ19-32 or GZ20-37 under hypoxic (1% O_2, 72 h) or normoxic (21% O_2, 72 h) conditions (column 1). Live cells were also incubated with the H7 antibody (CAIX antibody) and the secondary antibodies conjugated with Texas-Red (column 2) and the localization of the fluorescein and the CAIX antibody on cell surface is shown in column 3. The phase-contrast pictures are shown in column 4.

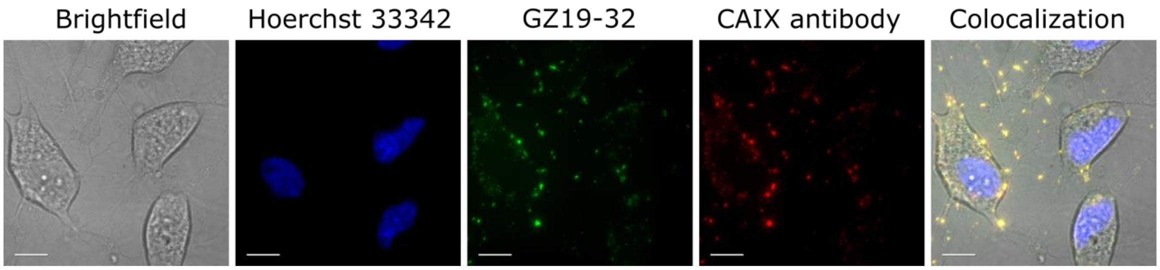

Figure S3. Application of lower concentration of GZ19-32 compound (2 nM) also yielded a well-co-localized positioning (fifth panel) of the compound (third panel, green) with the H7 antibody (fourth panel, red). The left panel shows the brightfield view of the cells. Both the compound and antibody were co-localized on the cell surface and the threads connecting the growing HeLa cells (yellow color, right panel). The scale bar length is 10 µm.

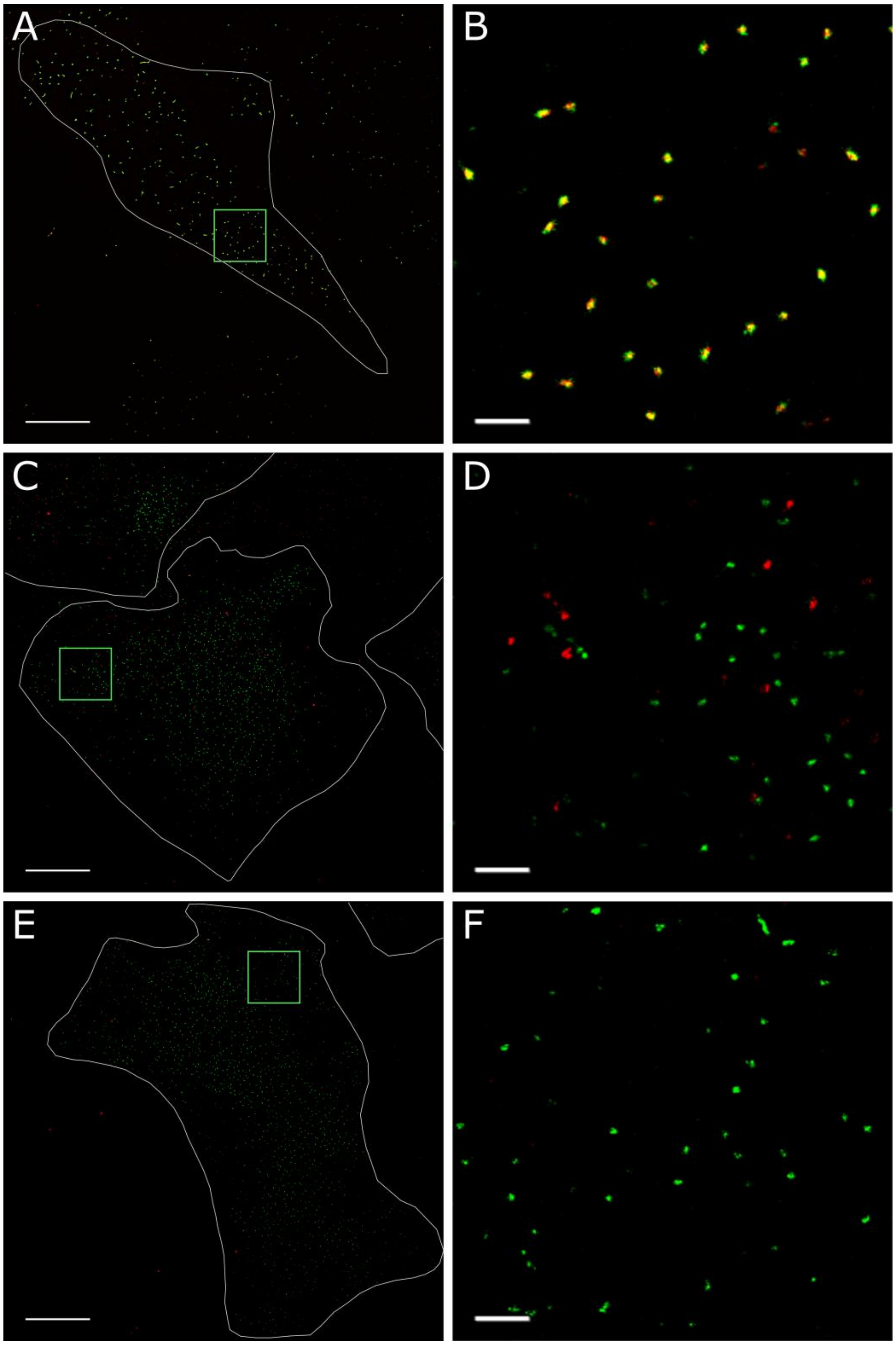

Figure S4. The basolateral plasma membrane of live HeLa cells, grown under hypoxia, are shown at super-resolution visualized by SRRF microscopy with TIRF illumination and illustrate the co-localization of the GZ19-32 (10 nM, green) with Alexa-594 labeled secondary anti-mouse antibody (1:100 dilution, red). **A**, **B**. The primary, CAIX-selective, M75 antibody was added. **C**, **D**. The primary, CAXII-selective, 1B10 antibody was added. The Pearson co-localization coefficient for M75 was 0.64, while for 1B10 was only 0.02. **E**, **F**. No primary antibody was added. In **A**, **C**, and **E** cell borders are shown with white lines and zoomed regions of interest with green squares are shown in **B**, **D**, and **F**. Scale bars are 10 µm in **A**, **C**, and **F** and 1 µm in **B**, **D**, and **F**.

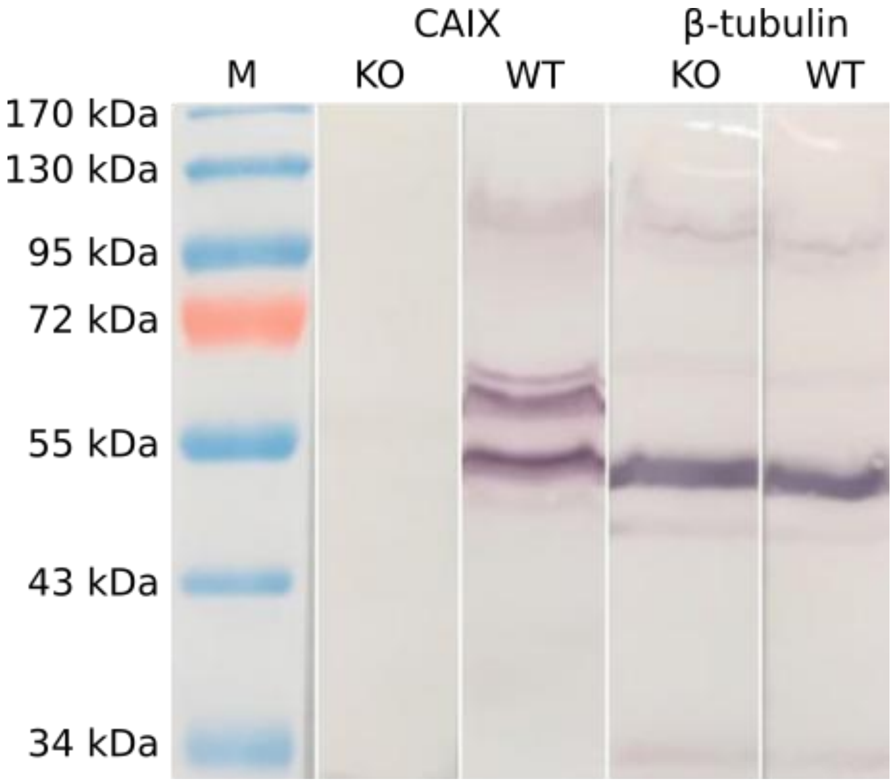

Figure S5. Full length gels of the WB described in Figure 6 and in the Materials and Methods section.

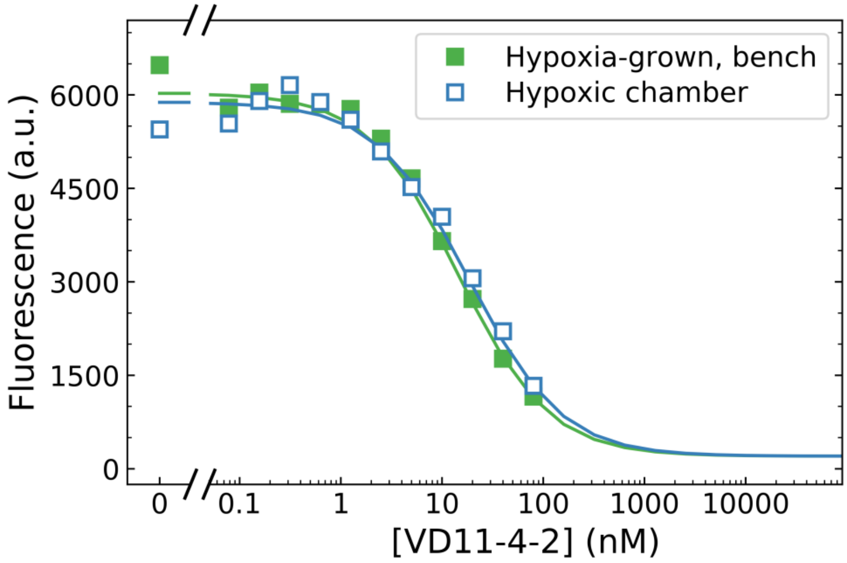

Figure S6. Comparison of the dosing curves of VD11-4-2 compound competition with 10 nM GZ19-32 obtained when the cells have been grown under hypoxia and the experiment of binding performed under normoxia for approximately 3 hours (filled green squares) and the cells were grown under hypoxia, and the whole competition experiment performed fully in hypoxic chamber (open blue squares).

Figure S7. Examples of compound NMR spectra.

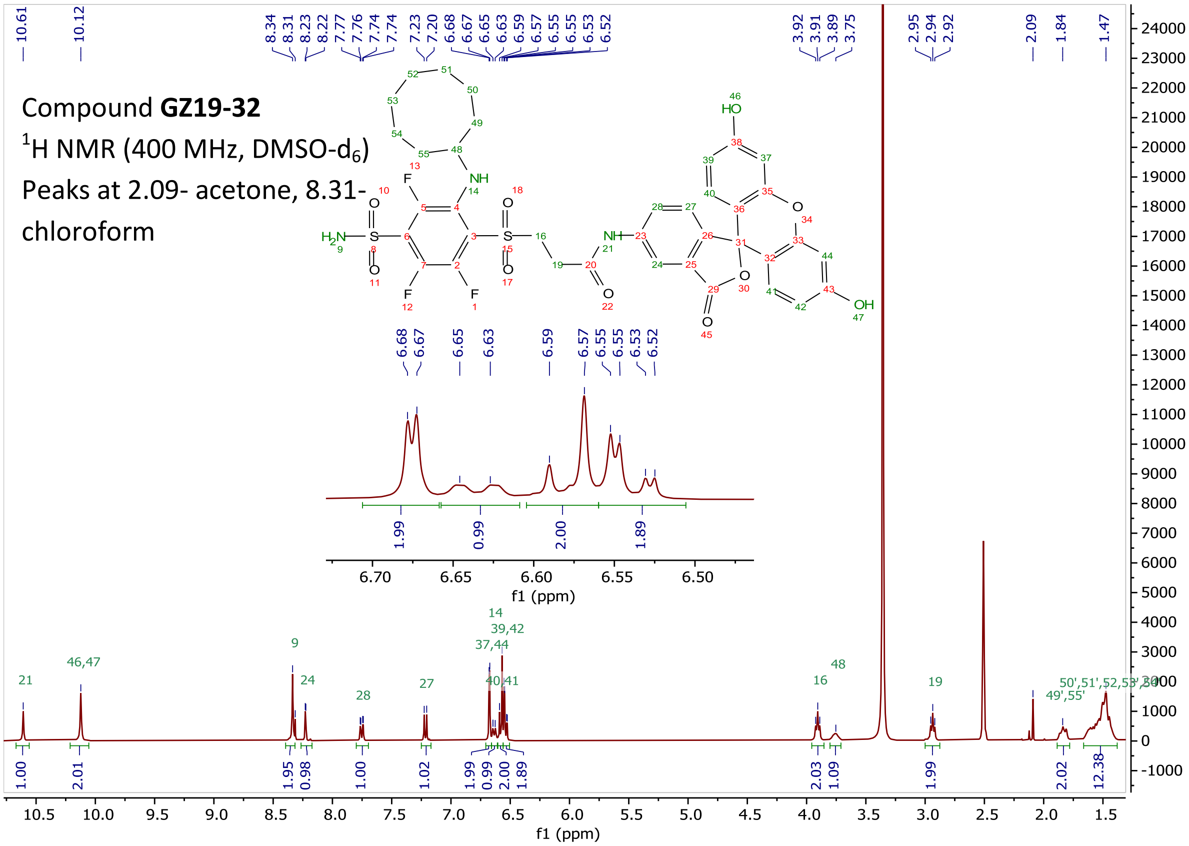

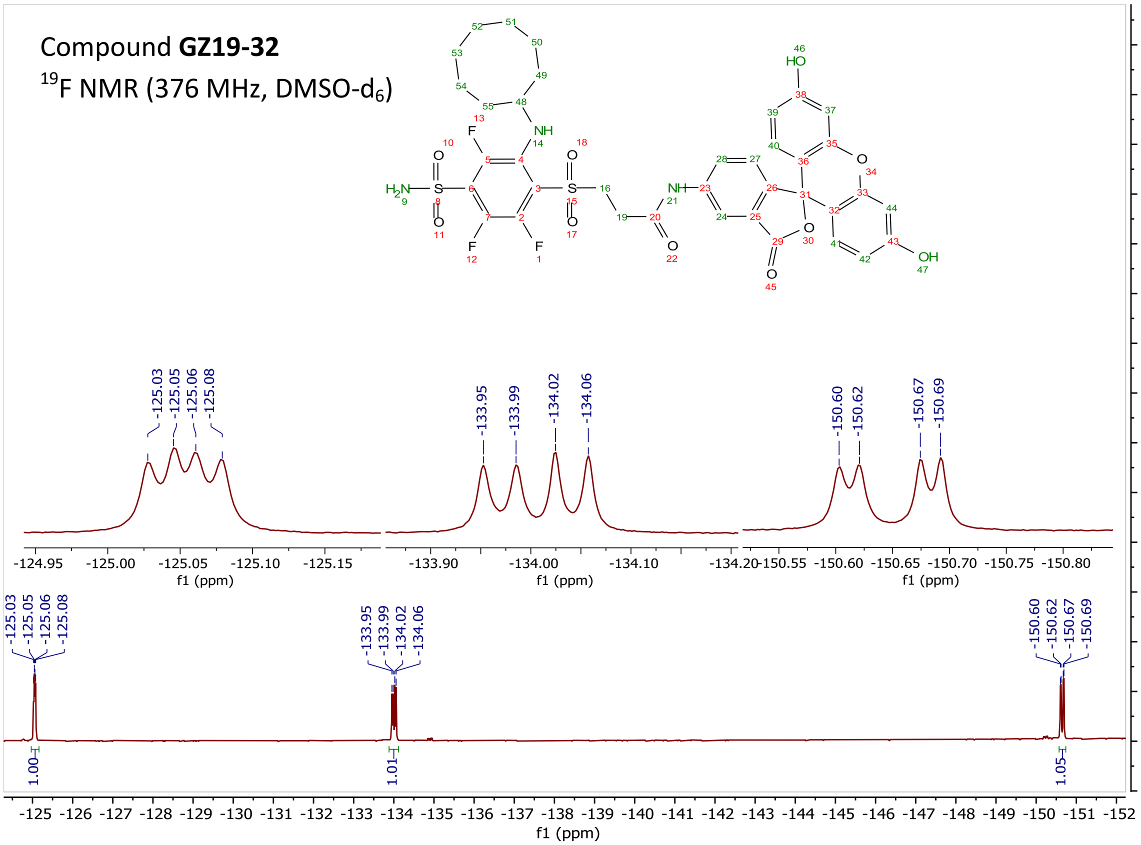
Compound **GZ19-32**
^{19}F NMR (376 MHz, DMSO-d$_6$)

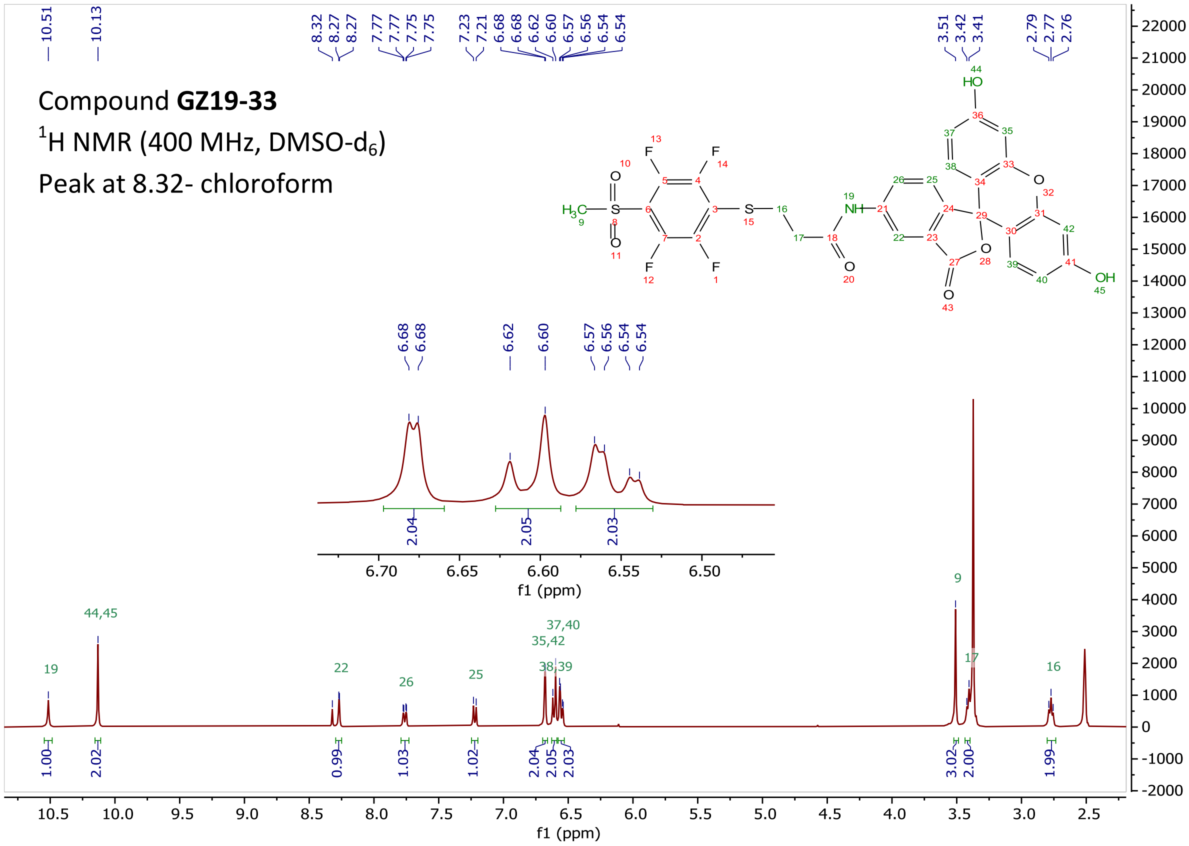
Compound **GZ19-33**
^{1}H NMR (400 MHz, DMSO-d$_6$)
Peak at 8.32- chloroform

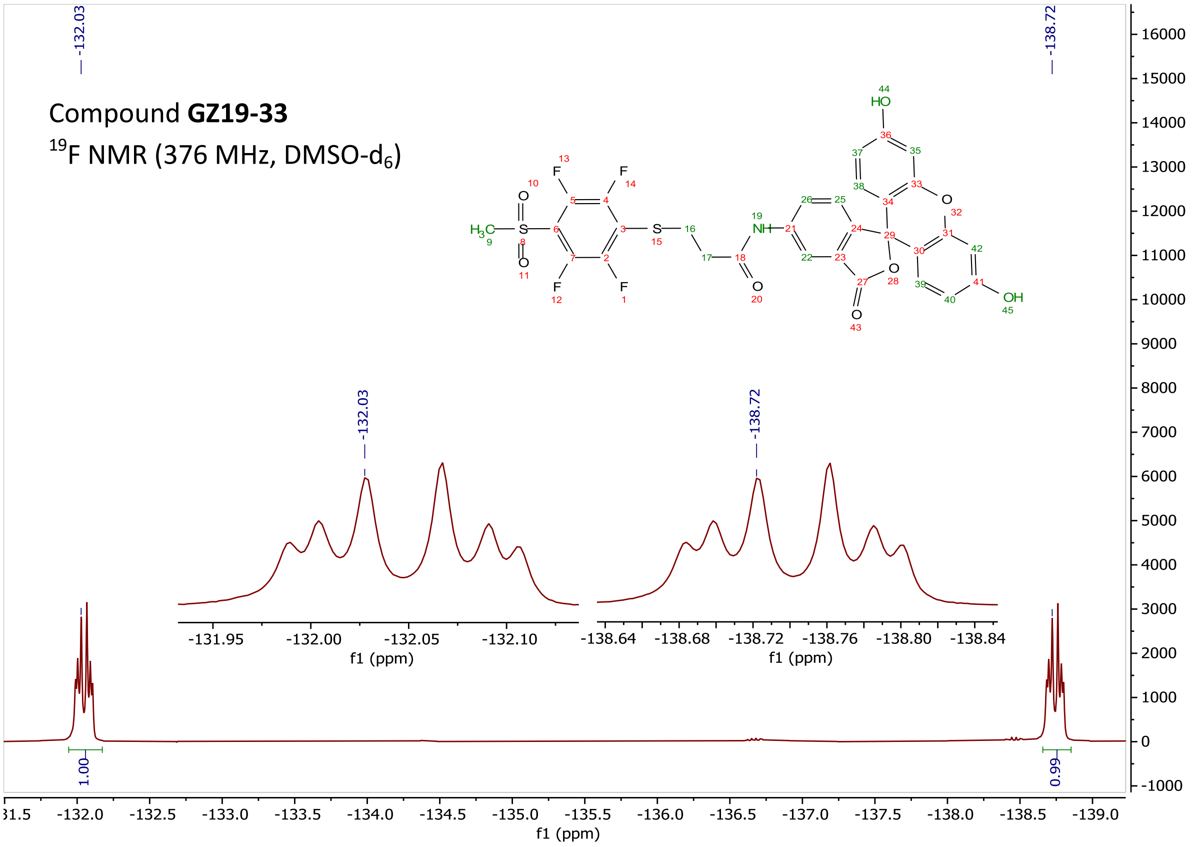

Compound **GZ19-33**
^{19}F NMR (376 MHz, DMSO-d$_6$)
-132.03
-138.72
1.00
0.99
f1 (ppm)
